# Influence of the Active Layer Structure on the Photovoltaic Performance of Water-Soluble Polythiophene-Based Solar Cells

**DOI:** 10.3390/polym13101640

**Published:** 2021-05-18

**Authors:** Massimiliano Lanzi, Debora Quadretti, Martina Marinelli, Yasamin Ziai, Elisabetta Salatelli, Filippo Pierini

**Affiliations:** 1 Department of Industrial Chemistry “Toso Montanari”, University of Bologna, Viale Risorgimento 4, 40136 Bologna, Italy; debora.quadretti2@unibo.it (D.Q.); martina.marinelli5@unibo.it (M.M.); elisabetta.salatelli@unibo.it (E.S.); 2 INSTM-National Interuniversity Consortium of Materials Science and Technology, Via G. Giusti 9, 50121 Firenze, Italy; 3 Department of Biosystem and Soft Matter, Institute of Fundamental Technological Research, IPPT-PAN, Polish Academy of Science, ul. Pawinskiego 5B, 02-106 Warsaw, Poland; yziai@ippt.pan.pl (Y.Z.); fpierini@ippt.pan.pl (F.P.)

**Keywords:** water-soluble polymers, double-cable copolymers, polythiophenes, GRIM polymerization, tributylphosphine, water-soluble fullerenes, OPVs

## Abstract

A new side-chain C_60_-fullerene functionalized thiophene copolymer bearing tributylphosphine-substituted hexylic lateral groups was successfully synthesized by means of a fast and effective post-polymerization reaction on a regioregular ω-alkylbrominated polymeric precursor. The growth of the polymeric intermediate was followed by NMR spectrometry in order to determine the most convenient reaction time. The obtained copolymer was soluble in water and polar solvents and was used as a photoactive layer in single-material organic photovoltaic (OPV) solar cells. The copolymer photovoltaic efficiency was compared with that of an OPV cell containing a water-soluble polythiophenic homopolymer, functionalized with the same tributylphosphine-substituted hexylic side chains, in a blend with a water-soluble C_60_-fullerene derivative. The use of a water-soluble double-cable copolymer made it possible to enhance the control on the nanomorphology of the active blend, thus reducing phase-segregation phenomena, as well as the macroscale separation between the electron acceptor and donor components. Indeed, the power conversion efficiency of OPV cells based on a single material was higher than that obtained with the classical architecture, involving the presence of two distinct ED and EA materials (PCE: 3.11% vs. 2.29%, respectively). Moreover, the synthetic procedure adopted to obtain single material-based cells is more straightforward and easier than that used for the preparation of the homopolymer-based BHJ solar cell, thus making it possible to completely avoid the long synthetic pathway which is required to prepare water-soluble fullerene derivatives.

## 1. Introduction

Conjugated polymer-based organic photovoltaic (OPV) solar cells are devices that can convert sunlight into electrical power via a multistep process: the generation of charged carriers upon light absorption, subsequent separation, transport, and collection to the respective electrodes [1]. Over the past decade, these devices have been widely studied [2] for their highly valuable features such as light weight, flexibility, and potentially low cost.

Among the different types of OPV cells, i.e., single layer and multilayer, the bulk-heterojunction (BHJ) cells are undoubtedly the most attractive due to their ability to combine high efficiency with easy preparation [3]. Indeed, in the BHJ architecture, the active layer—which is placed between an anode electrode (ITO) and a cathode (layer of Al)—is essentially formed by intermixing an electron donor (ED, a π-conjugated polymer) with an electron acceptor (EA, usually a fullerene derivative) material. The obtained heterojunction provides a high interface area between the two components, while promoting an efficient generation and transport of free-charge carriers. On the other hand, the phase separation that usually occurs with this type of system does not permit an ideal transport of charges and affects the blend morphology and its thermal stability, thus resulting in an important efficiency-limiting factor. Moreover, the commonly reduced miscibility of the two components is also an essential factor to be taken into account.

In order to obtain optimized and enhanced structures, a possible technique may be the chemical modification of the polymeric structure: a double-cable material is obtained by linking the EA group to the end of the side chains linked to the polyconjugated backbone [4]. However, even though important improvements such as a better morphology of the photoactive layer and a more efficient energy conversion of final devices have been obtained, earlier studies [5] have proved that the content of the EA group, e.g., fullerene, should be appropriate to obtain a good solubility in common organic solvents and at the same time an efficient collection of electrons during charge separation. Moreover, it should be recognized that the production of OPVs very often requires the consumption of large amounts of chlorinated and/or aromatic organic toxic solvents. Indeed, next generation photovoltaic technologies are clearly needed to make progress toward the study of large-scale environmentally friendly techniques.

In this context, due to the effective combination of excellent intrinsic optoelectronic properties [6] with a unique solubility in green solvents, water/alcohol-soluble conjugated polymers (WSCPs) have attracted growing attention in recent years [7,8,9,10]. Water solubility of π-conjugated polymers, in particular poly(3-alkylthiophene)s, can be achieved and tailored by incorporating hydrophilic ionic moieties—such as imidazolium [11,12], amino [13], or phosphine groups [14]—to monomer and/or polymer side chains to obtain conjugated polyelectrolytes (CPEs).

In addition to the use of WSCPs as electron donor materials, in order to prepare completely green active layers of BHJ cells and thus promote a high contact area between EA and ED components, water-soluble fullerene derivatives should also be used as EA moieties [15]. There are several different methods to make fullerene-C_60_ soluble in polar and green solvents, such as by chemical modification through the insertion of functional moieties or by the incorporation of fullerenes into water-soluble supramolecular structures, as well as solvent exchange methods or long-term stirring of pure C_60_ in water [16]. However, due to its high tendency to form aggregates in water, the main approach is to synthesize fullerene derivatives by attaching water-soluble groups, such as hydroxyl [17] and malonic acid derivatives [18], to the surface of C_60_.

**Scheme 1:**
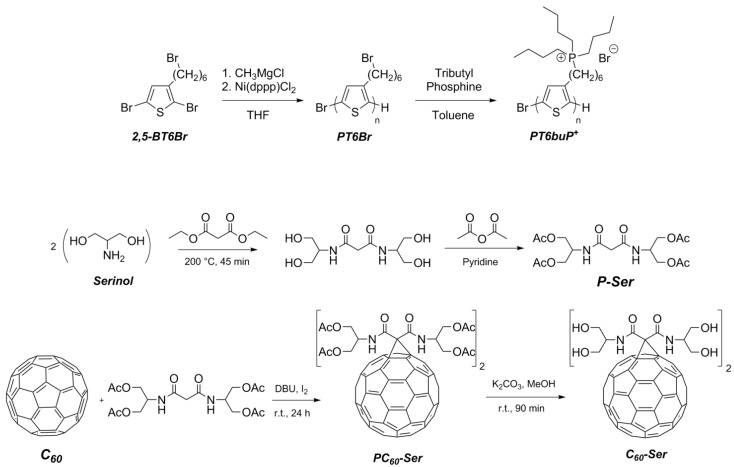
Synthetic route to obtain homopolymers (PT6Br and PT6buP^+^) and fullerene derivative (C_60_-Ser).

**Scheme 2:**
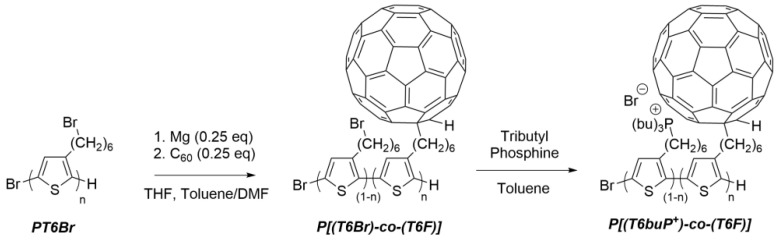
Synthesis of the water-soluble double-cable copolymer (P[T6buP^+^)-*co*-(T6F).

Therefore, the main aim of this work is to compare the photoconversion efficiency (PCE) of a water-soluble BHJ blend—composed by a serinol-fullerene derivative (C_60_-Ser) and an ionic homopolymer (PT6buP^+^)—with that of a new water-soluble double-cable copolymer bearing both a C_60_-fullerene moiety and an ionic phosphonium group at the end of a hexamethylenic side chain (P[(T6buP^+^)-*co*-(T6F)]). The adopted synthetic pathways are reported in Scheme 1 and Scheme 2.

The structural and photophysical properties of the synthesized materials were investigated using nuclear magnetic resonance (NMR), infrared (FT-IR) and ultraviolet–visible (UV–Vis) spectroscopy, thermogravimetric analysis (TGA), differential scanning calorimetry (DSC), X-ray diffraction, atomic force microscopy (AFM), and external quantum efficiency (EQE). The final materials were tested as active media in organic solar devices prepared with halogen-free solvents, obtaining the best photoconversion efficiency value (PCE 3.11%) using the double-cable copolymer.

## 2. Materials and Methods

### 2.1. Materials

[1,3-bis(diphenylphosphino)propane] nickel (II) chloride (Ni(dppp)Cl_2_), tributylphosphine (97%), methylmagnesium chloride (CH_3_MgCl, 3.0 M solution in THF), fullerene (C_60_, 98%), *N*,*N*-dimethylformamide (DMF, 99.8%), tetrahydrofuran (THF, >99.9%), toluene (C_7_H_8_, >99.9%), diethyl ether (Et_2_O, >99.5%), ethyl acetate (EtOAc, >99.5%), pyridine (>99.0%) and chloroform (CHCl_3_, 99.0–99.4%) were purchased from Sigma Aldrich Chemical Co. (St. Louis, Missouri, USA). *n*-Heptane (99.4%) was purchased from VWR Chemicals (Strasbourg, France). Methanol (MeOH, >99.8%) was purchased from Honeywell (Hanover, Germany). 1,8-Diazabicyclo [5.4.0]undec-7-ene (1,5–5) (DBU, >99%) was purchased from Fluka Chemika (Buchs, Switzerland). 2-Amino-1,3-propanediol (Serinol, >98.0%) was purchased from TCI (Tokyo, Japan). Diethyl malonate (99%) and acetic anhydride (Ac_2_O, 99%) were purchased from Carlo Erba (Milano, Italy). Iodine resublimed (I_2_) and magnesium (Mg) for Grignard were purchased from Merck (Darmstadt, Germany). 2,5-Dibromo-3-(6-bromohexyl)thiophene (2,5-BT6Br), as the starting monomer, was synthesized according to the method reported in the literature [19,20]. Just before use, the solvent used for the Grignard Metathesis Polymerization (GRIM) was dried with a sodium–potassium alloy under reflux and stored over molecular sieves. Diethyl ether and THF were freshly distilled before use to remove the stabilizer. All other materials were used as received. All manipulations involving moisture-sensitive reagents were performed under argon atmosphere in flame-dried glassware.

### 2.2. Synthesis of Homopolymers

#### 2.2.1. Poly[3-(6-bromohexyl)thiophene] (PT6Br) by Grignard Metathesis Polymerization (GRIM)

A total of 1.98 mL of a 3.0 M CH_3_MgCl solution (5.94 mmol) in THF was added to 2.40 g (5.94 mmol) of 2,5-BT6Br in 50.0 mL of anhydrous THF. The mixture was refluxed for 2 h under stirring in an inert atmosphere. The system was then cooled down to room temperature and 0.015 g (0.0297 mmol) of [1,3-bis(diphenylphosphino)propane] Nickel (II) chloride (Ni(dppp)Cl_2_) was added. Two portions of the mixture were taken out 30 and 60 min after the onset of the reaction, while the remaining part was stirred for 90 min. To stop polymerization, the three raw portions of the mixture were treated with HCl 2.5 M and extracted with chloroform. Organic phases were subsequently treated with HCl 1.0 M, washed with aqueous NaHCO_3_, and distilled water up to neutrality. Organic phases were dried over Na_2_SO_4_, filtered and concentrated to a small volume under reduced pressure.

The crude product obtained after 90 min of polymerization was precipitated into 900 mL of cold MeOH/HCl (1% HCl *v*/*v*) under stirring. The resulting dark brown powder was filtered on a cellulose extraction thimble (33 × 94 mm), washed with MeOH, and recovered with CHCl_3_ using a Soxhlet apparatus. The mixture was concentrated, reprecipitated in cold MeOH/HCl (0.2% HCl *v*/*v*), and then filtered on a PTFE membrane (0.45 μm pore size), giving 0.303 g (1.23 mmol, 21% yield) of reddish PT6Br.

PT6Br: ^1^H-NMR (400 MHz, CDCl_3_, ppm): δ 6.98 (s, 1H, Th *H*4), 3.43 (t, 2H, C*H*_2_Br), 2.82 (t, 2H, ThC*H*_2_), 1.95-1.84 (m, 2H, C*H*_2_CH_2_Br), 1.78-1.67 (m, 1H, ThCH_2_C*H*_2_), 1.58-1.40 (m, 4H, (C*H*_2_)_2_). ^13^C-NMR (100 MHz, CDCl_3_, ppm): δ 140.31 (Th *C*3), 134.91 (Th *C*5), 131.23 (Th *C*2), 129.01 (Th *C*4), 34.59 (*C*H_2_Br), 33.97, 30.61, 29.83, 29.17, 28.55 (aliphatic *C*H_2_). FT-IR (KBr, cm^−1^): 3058, 2923, 2852, 1508, 1434, 1384, 1259, 1089, 800, 725, 641, 558.

#### 2.2.2. Synthesis of Poly{3-[6-(tributylphosphonium)-hexyl]-thiophene-2,5-diyl Bromide} (PT6buP^+^) by Post-Functionalization of PT6Br

In a three-neck round-bottom flask, 0.200 g (0.816 mmol) of PT6Br was dissolved in 12.0 mL of toluene. Then, 2.0 mL (8.0 mmol) of tributylphosphine was added under stirring and in an inert atmosphere. The system was left to react at 90 °C for 24 h. The supernatant was removed and the polyelectrolyte formed, filmed on the flask surface, was recovered with methanol, washed with diethyl ether, and dried under vacuum to obtain 0.359 g (0.802 mmol, 98% yield) of PT6buP^+^ as a dark red solid.

PT6buP^+^: ^1^H-NMR (400 MHz, CD_3_OD, ppm): δ 7.13 (s, 1H, Th *H*4), 2.90 (bs, 2H, ThC*H*_2_), 2.24 (bt, 8H, C*H*_2_P^+^), 1.83-1.69 (bm, 2H, ThCH_2_C*H*_2_), 1.68-1.44 (bm, 18H, (C*H*_2_)_2_CH_2_P^+^), 0.98 (m, 9H, C*H*_3_). ^13^C-NMR (100 MHz, CD_3_OD, ppm): δ 141.64 (Th *C*3), 134.87 (Th *C*5), 131.63 (Th *C*2), 130.38 (Th *C*4), 31.42, 30.23, 29.67, 25.06, 24.91, 24.47, 22.46 (aliphatic *C*H_2_), 19.52 (*C*H_2_P^+^), 19.04 (P^+^*C*H_2_), 13.87 (*C*H_3_). ^31^P-NMR (400 MHz, CD_3_OD, ppm): δ 33.31. FT-IR (Ge, cm^−1^): 3053, 2957, 2929, 2870, 1513, 1463, 1410, 1378, 1232, 1099, 833, 723.

### 2.3. Synthesis of Water-Soluble Fullerene Derivative

#### 2.3.1. Synthesis of *N*,*N*’-Bis{2-(acetyloxy)-1-[(acetyloxy)methyl]ethyl}-malonamide (P-Ser)

In a loosely capped Pyrex tube, 0.98 g of serinol (2-amino-1,3-propanediol, 10.9 mmol) and 0.72 mL (4.57 mmol) of diethyl malonate were mixed and heated with vigorous stirring at 200 °C for 45 min. The heat was removed, EtOH was permitted to boil off, and the deep orange solid residue was treated with 4 mL of Ac_2_O and 4 mL of pyridine and stirred for 20 h at room temperature. A total of 3.0 mL of methanol was carefully added, while stirring and cooling the system to 0 °C. The solvents were removed under vacuum and the residue was recovered in 30 mL of EtOAc. The solid was then extracted with a diluted solution of CuSO_4_ (10^−3^ M, 3 × 20 mL), washed with distilled water (3 × 30 mL) and finally extracted with a saturated solution of NaCl. The organic phase was dried with Na_2_SO_4_ and filtered to give 0.631 g (1.51 mmol, 33% yield) of deep orange oil.

P-Ser: ^1^H-NMR (400 MHz, CDCl_3_, ppm): δ 7.34 (b, 2H, N*H*), 4.43 (m, 2H, C*H*NH), 4.16 (m, 8H, OC*H*_2_), 3.21 (s, 2H, COC*H*_2_CO), 2.07 (s, 12H, C*H*_3_). ^13^C-NMR (100 MHz, CDCl_3_, ppm): δ 170.91 (CH_3_*C*OO), 167.15 (*C*ONH), 62.65 (O*C*H_2_), 47.61 (*C*HNH), 42.64 (CO*C*H_2_CO), 20.89 (*C*H_3_). FT-IR (Ge, cm^−1^): 3301, 2961, 2902, 1737, 1661, 1537, 1370, 1242.

#### 2.3.2. Synthesis of Protected Malonodiserinolamide Fullerene (PC_60_-Ser)

In a two-neck round-bottom flask, 0.0644 g (0.0894 mmol) of fullerene (C_60_) was dissolved in 110 mL of anhydrous toluene. A total of 0.187 g (0.447 mmol) of P-Ser, 0.13 mL (0.894 mmol) of DBU and 0.113 g (0.447 mmol) of I_2_ were added to the system, and the mixture was left to react for 24 h under stirring, at room temperature and in an inert atmosphere.

The solution was washed repeatedly with distilled water (2 × 100 mL) and a saturated solution of NaCl (1 × 100 mL). After drying with Na_2_SO_4_ and concentrating at reduced pressure, 0.174 g (0.112 mmol, 25% yield) of PC_60_-Ser was obtained.

PC_60_-Ser: ^1^H-NMR (400 MHz, CDCl_3_, ppm): δ 7.39 (br d, J = 7.78 Hz, 2H, N*H*), 4.69 (m, 2H, C*H*NH), 4.35 (dd, 8H, OC*H*_2_), 2.09 (s, 12H, C*H*_3_). ^13^C-NMR (100 MHz, CDCl_3_, ppm): δ 171.01 (CH_3_*C*OO), 166.80 (*C*ONH), 143.30 (C_60_), 138.25, 131.57, 129.36, 128.55, 125.62, 125.45, 125.42, 73.19 (subst. C_60_), 62.78 (O*C*H_2_), 58.79 (OC*C*CO), 49.45 (*C*HNH), 21.11 (*C*H_3_). FT-IR (Ge, cm^−1^): 3289, 2926, 2853, 1739, 1646, 1543, 1428, 1366, 1228, 1880, 561, 526.

#### 2.3.3. Synthesis of Malonodiserinolamide Fullerene (C_60_-Ser)

A total of 0.100 g (0.0644 mmol) of PC_60_-Ser was added to 40 mL of MeOH and 40 mL of a saturated solution of K_2_CO_3_. The mixture was left to react under stirring at room temperature for 90 min. The organic phase was removed, and the dark brown solid was dried under reduced pressure. The solid was washed several times with methanol, filtered on a PTFE membrane (0.45 μm pore size) and finally dried, giving 0.0678 g (0.0557 mmol, 87% yield) of the final product.

C_60_-Ser: ^1^H-NMR (400 MHz, CD_3_OD, ppm): δ 3.73 (m, 4H, C*H*_2_OH), 3.48 (m, 4H, C*H*_2_OH), 3.13 (m, 2H, C*H*NH). ^13^C-NMR (100 MHz, CD_3_OD, ppm): δ 161.42 (*C*ONH), 141.00 (C_60_), 73.04 (subst. C_60_), 52.72 (HO*C*H_2_), 52.50 (CO*C*CO), 52.28 (*C*HNH). FT-IR (KBr, cm^−1^): 3405, 2948, 1649, 1429, 1401, 1371, 1182, 576, 526.

### 2.4. Synthesis of Water-Soluble Double-Cable Copolymer

#### 2.4.1. Synthesis of Poly [3-(6-Bromohexyl)thiophene-co-3-(6-fullerenylhexyl)thiophene] (P[(T6Br)-co-(T6F)]) by Post-Functionalization of PT6Br

A total of 0.103 g (0.420 mmol) of PT6Br and 0.0026 g (0.105 mmol) of Mg were added into 5.0 mL of anhydrous THF in the first reaction system. The mixture was refluxed for 2 h under stirring and in an inert atmosphere. Then, the system was cooled down to room temperature, and the crude reaction mixture was transferred to the second reaction system containing 1 mL of DMF, 100 mL of anhydrous toluene and 0.114 g (0.158 mmol) of C_60_. The mixture was left to react at room temperature for 22 h under vigorous stirring. Subsequently, 1 mL of a solution of NH_4_Cl (2 M) in distilled water was added to quench the reaction mixture. The crude product was extracted with a half-saturated solution of NaCl (2 × 100 mL) and washed with distilled water (2 × 100 mL). The organic phase was dried under vacuum, and the obtained solid was recovered with 50 mL of CHCl_3_ and slowly added to 350 mL of n-heptane. The dark brown precipitate was filtered on a PTFE membrane (0.45 μm pore size) and dried, giving 0.104 g (0.357 mmol, 85% yield) of copolymer.

P[(T6Br)-*co*-(T6F)]: ^1^H-NMR (400 MHz, pyridine-d_5_, ppm): δ 7.45 (s, Th *H*4), 7.41 (s, C_60_-*H*), 3.57 (bm, C*H*_2_C_60_), 3.45 (bt, C*H*_2_Br), 3.01 (bt, ThC*H*_2_), 1.88-1.66 (bm, C*H*_2_CH_2_Br + C*H*_2_CH_2_C_60_), 1.54-1.32 (bm, ThCH_2_C*H*_2_), 1.32-1.12 (bm, (C*H*_2_)_2_CH_2_CH_2_Br + (C*H*_2_)_2_CH_2_CH_2_C_60_). ^13^C-NMR (100 MHz, pyridine-d_5_, ppm): δ 143.13 (*C*_60_), 134.99, 122.96 (Thiophene *C*), 60.05 (*C*H_2_C_60_), 32.03 (*C*H_2_Br), 29.20, 22.89 (aliphatic *C*H_2_). FT-IR (KBr, cm^−1^): 3053, 2918, 2849, 1509, 1452, 1428, 1384, 1257, 1181, 1099, 826, 722, 644, 557, 562, 526.

#### 2.4.2. Synthesis of Poly [3-(6-Tributylphosphonium)thiophene-co-3-(6-fullerenylhexyl)thiophene] (P[(T6buP^+^)-co-(T6F)]) by Post-Functionalization of P[(T6Br)-co-(T6F)]) with Tributylphosphine

A total of 0.100 g (0.323 mmol) of P[(T6Br)-*co*-(T6F)] was mixed with 0.570 mL (2.27 mmol) of tributylphosphine in 5.0 mL of toluene. The mixture was left to react at 90 °C for 5 h under stirring and in an inert atmosphere. The system was cooled down to room temperature and the supernatant was removed. The dark brown-reddish solid was recovered in 30.0 mL of MeOH, washed with diethyl ether and dried under vacuum, giving 0.121 g (0.252 mmol, 78% yield) of copolymer.

P[(T6buP^+^)-*co*-(T6F)]: ^1^H-NMR (400 MHz, pyridine-d_5_, ppm): δ 7.54 (s, Th *H*4), 7.47 (s, C_60_-*H*), 3.56 (bm, C*H*_2_C_60_), 3.07 (bm, ThC*H*_2_(CH_2_)_5_C_60_), 2.92 (bm, ThC*H*_2_(CH_2_)_5_P^+^), 2.79 (bm, C*H*_2_P^+^), 1.94-1.82 (bm, ThCH_2_C*H*_2_(CH_2_)_4_P^+^ + C*H*_2_CH_2_C_60_), 1.82-1.12 (m, (C*H*_2_)_3_CH_2_P^+^ + (C*H*_2_)_3_CH_2_CH_2_C_60_), 1.04-0.81 (bm, C*H*_3_). ^13^C-NMR (100 MHz, pyridine-d_5_, ppm): δ 143.11 (*C*_60_), 134.97, 122.95 (Thiophene *C*), 60.12 (*C*H_2_C_60_), 28.59, 27.95, 24.42, 24.34, 24.30, 24.25, 24.19, 23.96 (aliphatic *C*H_2_), 19.37 (*C*H_2_P^+^), 18.90 (P^+^*C*H_2_), 13.80 (*C*H_3_). ^31^P-NMR (400 MHz, CD_3_OD, ppm): δ 37.84. FT-IR (Ge, cm^−1^): 3052, 2956, 2927, 2869, 1514, 1461, 1427, 1408, 1379, 1228, 1180, 1096, 808, 721, 576, 526.

### 2.5. Measurements

^1^H-NMR, ^13^C-NMR and ^31^P-NMR were recorded on a Varian Mercury Plus 400 spectrometer using TMS as a reference. IR spectra were taken on Ge or KBr disks using a Perkin Elmer Spectrum One spectrophotometer. UV–Vis spectra were recorded on a Perkin Elmer Lambda 19 spectrophotometer using either around 10^−5^ M polymer solutions in spectroquality solvents in Suprasil quartz cuvettes (1 × 1 cm) or films on quartz slides. The molecular weight was determined by gel permeation chromatography (GPC) using THF solutions on a HPLC Lab Flow 2000 apparatus equipped with a Rheodyne 7725i injector, a Phenomenex MXL 5 μm mixed bed column and an RI K-2301 KNAUER detector. The calibration curve was recorded using monodisperse polystyrene standards. A TA Instruments Q2000 differential scanning calorimeter (DSC) was used for the thermal analysis by varying the temperature from −50 to 200 °C with a heating and cooling ramp of 10 °C min^−1^ in a nitrogen atmosphere. A TA Instruments Q600 thermogravimetric analyzer (TGA), operating in air flux, was used to determine sample decomposition temperatures by heating from 30 °C to 600 °C with a ramp of 10 °C min^1^. X-ray diffraction data were recorded at room temperature by using a CuKα (λ = 1.5406 Å) radiation source (Philips PW 1050) and a Bragg–Brentano diffractometer (Philips PW 1710) equipped with a graphite monochromator in the diffracted beam. The 2θ range between 2.0 and 90.0° was scanned by 881 steps of 0.1° with a counting time of 15 s for each step. The XRD characterization was carried out on polymer films on glass slides deposited by doctor blading (100 nm average thickness) after an annealing procedure (heating at 120 °C for 45 min under vacuum). Field emission scanning electron microscopy (FE-SEM) was performed with a FEI Nova NanoSEM 450 microscope at an accelerating voltage of 10 kV and a working distance of about 4 mm. Samples were covered with an 8 nm-thick gold layer, using a SC7620 Polaron mini sputter coater (Quorum Technologies Ltd., Ashford, UK) before performing the electron microscopy imaging. The sample surface topography was evaluated with an atomic force microscope (AFM, Ntegra, NT-MDT) equipped with a silicon cantilever (HA_NC, NT-MDT, tip radius of 10 nm, and spring constant of 12 N/m). Measurements were performed in a semi-contact mode with a resonance frequency of around 150 kHz; the scanning rate was 0.5 Hz, while the size of the scanned sample was 2.75 µm × 2.75 µm per image.

### 2.6. Solar Cells

BHJ solar cells were prepared according to the following procedure: the ITO glass substrate (2 × 2 cm, surface resistance 21 Ω/sq) was etched on one side by using a 10% wt aqueous solution of HCl, and heated at 60 °C for 10 min, in order to obtain an area of 1.5 × 1 cm covered by indium tin oxide. The glass was then cleaned using distilled water, 2-propanol and dried with a nitrogen flow. The final resistance of the ITO glass was 12 Ω/sq. Poly(3,4-ethylenedioxythiophene):polystyrene sulfonic acid (PEDOT:PSS, 2.8 wt% dispersion in water, viscosity 20 cps) was diluted 1:1 *v*/*v* with 2-propanol, sonicated for 15 min using an ultrasonic bath (Elmasonic S 30H), filtered on a Gooch G2, and the resulting solution (viscosity 12 cps) deposited over the previously treated ITO glass with the doctor blading technique using a Sheen Instrument Model S265674, leaving only a small (0.5 × 1 cm) area uncovered at the opposite side of the previously etched area.

The PEDOT:PSS film was heated in a Buchi GKR-50 glass oven at 120 °C for 90 min under vacuum (10^−3^ mmHg). A solution made by mixing (i) 2.5 mg of PT6buP^+^ and 2.5 mg of C_60_-Ser in 0.5 mL of methanol and (ii) 5 mg of P[(T6buP^+^)-*co*-(T6F)] in 0.5 mL of methanol was sonicated for 15 min and deposited by doctor blading on the slide in order to cover the PEDOT:PSS layer. The sample was then annealed in the glass oven under vacuum (10^−3^ mmHg) at 120 °C for 45 min. Finally, a 50 nm-thick Al electrode was deposited over the polymeric layer through a shadow mask using an Edwards 6306A coating system operating at 10^−6^ mmHg. The active area of the cell was 1 × 1 cm^2^. The current–voltage characteristics were measured in air using a Keithley 2401 source meter under the illumination of an Abet Technologies LS150 Xenon Arc Lamp Source AM 1.5 Solar Simulator (100 mW/cm^2^) calibrated with an ILT 1400-BL photometer. The structure of the final devices was made of: ITO (80 nm)/PEDOT:PSS (100 nm)/active layer (150 nm)/Al (50 nm). The solar cell spectral response was measured using a 7-SC Spec III Modularized Solar Cell Spectral Test System (SevenStar Optics, Beijing, Chian). Layer thickness was measured using a FTPAdvances FTPadv-2 Film Thickness Probe (Sentech Instruments GmbH, Berlin, Germany) equipped with the FTPExpert software.

## 3. Results and Discussion

### 3.1. Synthesis

The regioregular homopolymeric precursor PT6Br was prepared starting from 2,5-dibromo-3-(6-bromohexyl)thiophene (2,5-BT6Br) [5,20] (p. 2,3); while the post-polymerization functionalization of PT6Br gave the corresponding water-soluble homopolymer PT6buP^+^, the water-soluble electron acceptor fullerene derivative (C_60_-Ser) was obtained from the properly functionalized serinol, according to the procedure shown in Scheme 1.

In detail, since a high value of regioregularity is the first key point to improve the final optical and conductive properties of the photoactive material, the regiospecific GRIM (Grignard Metathesis) [21,22,23] polymerization method was adopted to obtain a regioregular PT6Br with a high percentage of HT linkages (95%). The synthesis is based on the formation of a Grignard organomagnesium intermediate which is obtained by first treating the dibrominated monomer with CH_3_MgCl under reflux, and its subsequent cross-coupling reaction with Ni(dppp)Cl_2_ as a catalyst. To determine the optimal reaction time (90 min), the degree of polymerization after the addition of the catalyst was followed by quenching two portions of the reaction mixture after 30 and 60 min.

A greater solubility in water of the polymers with cationic functionalities is usually observed than with anionic groups [8] (p. 2); therefore, the ionic water-soluble homopolymer—poly[3-(6-tributylphosponium-hexyl)thiophene bromide] (PT6buP^+^)—was obtained by a substitution reaction on the bromoalkylic side chain of the non-ionic regioregular homopolymeric precursor PT6Br. Indeed, the reaction with phosphines generally makes it possible to obtain the complete post-functionalization of side chain substituents [13] (p. 2), thus increasing the solubility of PT6buP^+^ in water (35 mg/mL) and polar solvents (up to 50 mg/mL in MeOH). Moreover, since a post-functionalization procedure has been chosen, the regioregularity degree of the ionic functionalized polythiophene is pre-determined in the polymeric precursor (95% HT dyads), and then the configurational regularity of the resulting polymer cannot be affected by the substitution reaction. The ionization of bromide groups takes place under reflux, in the presence of an excess of tributylphosphine (1:10 eq) and toluene as a solvent, to promote the SN_2_ reaction on the bromide group. Then, the formed polyelectrolyte, which is insoluble in common organic solvents such as toluene, tends to precipitate and can therefore be easily recovered from the reaction system.

The reduced solubility of fullerenes, which is limited to only a few organic solvents such as toluene and chlorinated aromatics, combined with the high hydrophobicity and tendency to form aggregates in solutions, has prompted recent studies [15] (p. 2) focusing on the chemical modification of fullerene (C_60_) by means of different synthetic approaches. Usually, the main electron acceptor material used in BHJ solar cells is PC_61_BM, a fullerene ester-derivative which, owing to its good solubility and miscibility with π-conjugated polymers, performs quite well when they are mixed together in a blend. Other fullerene-based materials such as fulleropyrrolidine, PC_71_BM, or Indene-C_60_ bis-adduct could also be used for the preparation of organic solar cells, but their different solubility has an impact on morphologies, and consequently on device efficiency. Indeed, the solvent used for the deposition of the blend, together with the composition, solution concentration, chemical structure, and control of phase separation, plays a key role in the film formation and, therefore, on the final device efficiency [24]. For this purpose, an electron acceptor material which is completely soluble in water and alcohols, like the previously prepared electron donor polymer (PT6buP^+^), was synthesized to overcome any miscibility problems with the two components in the final BHJ photoactive layer.

The synthesis approach adopted to make fullerene soluble in aqueous systems involves the nucleophilic addition of the protected serinolamide malonate to C_60_ by the modified Bingel–Hirsch reaction [25], as shown in Scheme 1. In detail, according to the paper of Wharton et al. [26], serinol and diethylmalonate were first condensed to give serinolamide, which was subsequently protected with acetic groups to give the corresponding ester. A different synthetic route—involving the use of I_2_ as a less toxic and easier-to-handle reagent [27] instead of Br_2_—was followed for the synthesis of the halogenated malonate intermediate. Indeed, the replacement of one [6-6] fullerene double bond through a cyclopropanation reaction requires the in situ synthesis of iodomalonate, in the presence of C_60_ and DBU as a base [28]. The excess of reagents with respect to fullerene was established with the aim of obtaining a bis-adduct since the deprotected mono-adduct is poorly soluble in water, as reported in Wharton’s work [29]. The final water solubility of the fullerene derivative was then achieved after basic hydrolysis, in order to restore hydroxyl groups.

The reduced distance between the EA and ED materials obtained by the direct insertion of a fullerene in the side chain of conjugated polymers could lead to an improved morphology and photoconversion efficiency of photovoltaic devices. For this purpose, the double-cable copolymer P[(T6buP^+^)-*co*-(T6F)] was prepared to combine the excellent solubility in water of phosphine-substituted polythiophene-based materials with the high electron affinity of fullerene derivatives. In particular, starting from the previously synthesized PT6Br, a non-ionic double-cable copolymeric precursor P[(T6Br)-*co*-(T6F)] with 7% fullerene content was prepared, according to the procedure reported in the literature [5] (p. 2). The obtained product was then post-functionalized with tributylphosphine using the same reaction conditions as the preparation of the homopolymer PT6buP^+^, but with a reduced reaction time (from 24 to 5 h), in order to prevent any loss of C_60_ from the side chains (Scheme 2). An overview and comparison of the main homo- and double-cable copolymers’ characteristics is reported in Table 1.

### 3.2. NMR Characterization

All the synthesized products were characterized by ^1^H-NMR and ^13^C-NMR spectrometry to evaluate their chemical structure and purity degree (see Supporting Information for ^1^H-NMR and ^13^C-NMR spectra).

**Figure 1:**
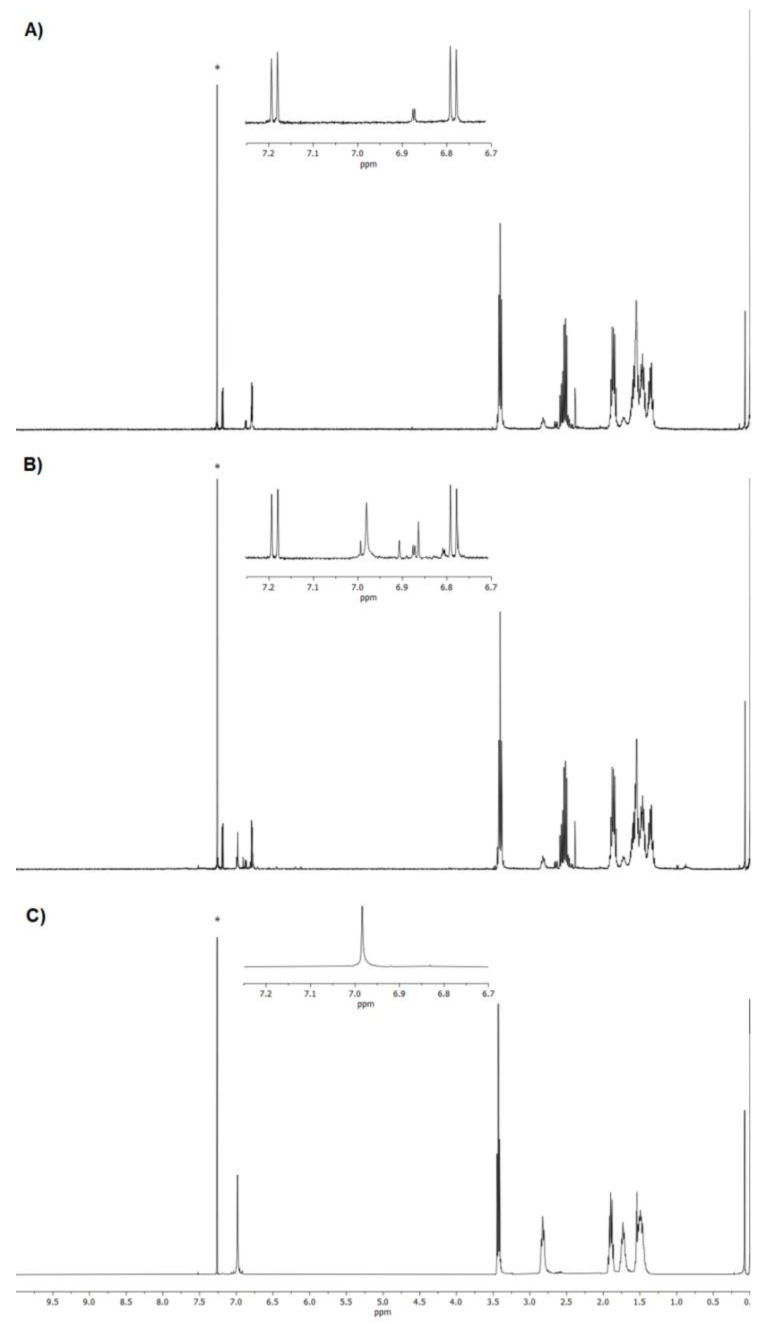
^1^H-NMR spectra of PT6Br after 30 min (**A**), 60 min (**B**) and 90 min (**C**). Asterisk: solvent resonance (CDCl_3_). Inset: expansion of the aromatic region.

The synthesis of the highly regioregular PT6Br with well-defined molecular weight by GRIM reaction, as a quasi-living polymerization [30], was also followed by ^1^H-NMR spectroscopy by collecting three reaction mixture samples at different reaction times (30, 60 and 90 min) after the addition of the Ni(dppp)Cl_2_ catalyst. Chemical shifts and assignments are shown in Table S1 [31,32,33,34,35,36], while the spectra are shown in Figure 1.

The ^1^H-NMR spectrum collected 30 min after the onset of the reaction (Figure 1A) essentially shows only the signals ascribable to reagents, i.e., the quenched isomers deriving from the Grignard intermediate. An analysis of the spectrum obtained after 60 min (Figure 1B) reveals a different situation: while Grignard intermediates are still clearly present, the peaks related to two or more thiophenic units that are linked one to another are also shown. The presence of two characteristic signals, at 6.81 ppm and 7.00 ppm—ascribable to the first TT linkage between the two starting thiophenic units and to the polymeric backbone growing with TT-HT linkages, respectively—suggests that the polymerization is still taking place in the reaction system. Indeed, after 90 min (Figure 1C), the exclusive presence of peaks related to the main polymeric chain—with HT linkages and bromide groups in the side chain—indicates that polymerization has occurred. In view of this, it is therefore possible to state that, in the case of ω-bromoalkylthiophene derivatives, the best reaction time for the second catalyzed step of the GRIM reaction at room temperature is 90 min.

^1^H-NMR spectroscopy was also used to estimate the percentage of head-to-tail linkages (95%) in the macromolecular backbone of the final PT6Br (Figure 1C), since the regioregularity degree can be evaluated by the integral ratio of the signals centered at 2.83 ppm (HT dyads) and 2.59 ppm (HH and TT dyads).

**Figure 2:**
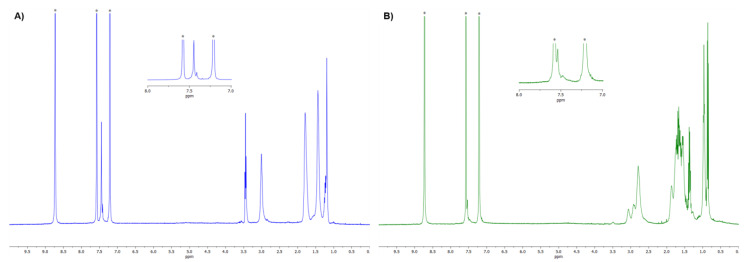
^1^H-NMR spectra of double-cable copolymers, P[(T6Br)-*co*-(T6F)] (blue line), (**A**) and P[(T6buP^+^)-*co*-(T6F)] (green line), (**B**). Asterisk: solvent resonance pyridine-d_5_. Inset: expansion of the aromatic region.

Similarly, it is also possible to confirm the successful post-functionalization of PT6Br with C_60_-fullerene to obtain the double-cable precursor copolymer P[(T6Br)-*co*-(T6F)]. Indeed, the copolymer spectrum (Figure 2A), registered in pyridine-d_5_, clearly shows the characteristic singlet peak at 7.41 ppm, as well as the broad multiplet at 3.56 ppm—ascribable to the fullerene proton and methylenic protons in the α-position to C_60_, respectively—that are only present when the fullerene is directly linked to the side chain. Going by the ratio of the signals at 3.56 and at 3.45 ppm, where the latter is related to the C*H*_2_Br of the side chain, a 7% fullerene content can be estimated for the precursor copolymer.

The occurrence of the post-functionalization with tributylphosphine of PT6Br and P[(T6Br)-*co*-(T6F)], to give PT6buP^+^ and P[(T6buP^+^)-*co*-(T6F)], respectively, was verified by both ^1^H-NMR and ^13^C-NMR, as well as ^31^P-NMR spectroscopy. In particular, when analyzing ^1^H-NMR spectra, the presence of a broad multiplet at 2.75 ppm (P[(T6buP^+^)-*co*-(T6F)] spectrum, Figure 2B) and a broad triplet at 2.24 ppm (PT6buP^+^ spectrum, Figure S1) can be attributed to the eight methylenic protons in α- and α’-position to the cationic phosphine group. In addition, the total absence of signals ascribable to –Br moieties evidences the total substitution reaction with phosphonium group for both materials, which typically occurs when phosphines are used as nucleophile reagents. Moreover, when ^13^C-NMR spectra of PT6buP^+^ and P[(T6buP^+^)-*co*-(T6F)] (Figures S2 and S3) are compared with those of the corresponding precursor polymers PT6Br and P[(T6Br)-*co*-(T6F)] (Figures S4 and S5), a further confirmation that the post-functionalization took place is obtained. In detail, passing from the PT6Br to PT6buP^+^ spectrum, the signal at 34.59 ppm (*C*H_2_Br) is missing, while three new signals appear at 19.52 (*C*H_2_P^+^), 19.04 (P^+^*C*H_2_), and 13.87 (*C*H_3_) ppm. A similar result is obtained from the comparison of the spectra of P[(T6Br)-*co*-(T6F)] and P[(T6buP^+^)-*co*-(T6F)]: in this case, the signal at 32.03 ppm (*C*H_2_Br) present in the brominated copolymer spectrum is completely missing in that of the water-soluble copolymer and replaced by the signals belonging to the tributylphosphonium group at 19.37 (*C*H_2_P^+^), 18.90 (P^+^*C*H_2_), and 13.80 (*C*H_3_) ppm. ^31^P-NMR spectra of PT6buP^+^ and P[(T6buP^+^)-*co*-(T6F)] (Figure S6) also confirm the complete absence of unreacted tributylphosphine, as previously verified by ^1^H-NMR.

Finally, the obtainment of the precursors of C_60_-Ser (i.e., P-Ser and PC_60_-Ser) and water-soluble fullerenic derivative C_60_-Ser has also been confirmed by ^1^H-NMR (Figures S7–S9) and ^13^C-NMR (Figures S10–S12) spectrometry.

In detail, the absence of the singlet at 3.21 ppm ascribable to the COC*H*_2_CO protons of the protected serinol (P-Ser) in the PC_60_-Ser spectrum clearly indicates that the expected reaction on the fullerene surface has occurred. This is also confirmed by the presence of the signals at 143.30 (C_60_) and 73.19 ppm (substituted C_60_) in the PC_60_-Ser ^13^C-NMR spectrum. Then, the successful deprotection of malonic groups to give C_60_-Ser is confirmed by the absence of the signals at 4.35 (OC*H*_2_) and 2.09 (*C*H_3_) ppm ascribable to the acetoxy group protons in the side chains. As for PC_60_-Ser, the presence of fullerene directly linked to a malonamide derivative is indicated by the signals at 141.00 and 73.04 ppm in the C_60_-Ser ^13^C-NMR spectrum.

### 3.3. FT-IR Characterization

FT-IR spectroscopy further confirms the composition of all synthesized materials. The FT-IR bands of homopolymers (PT6Br and PT6buP^+^), double-cable copolymers (P[(T6Br)-*co*-(T6F)] and P[(T6buP^+^)-*co*-(T6F)]) and electron acceptor material (C_60_-Ser), as well as their assignments, are shown in Tables S2 and S3.

**Figure 3:**
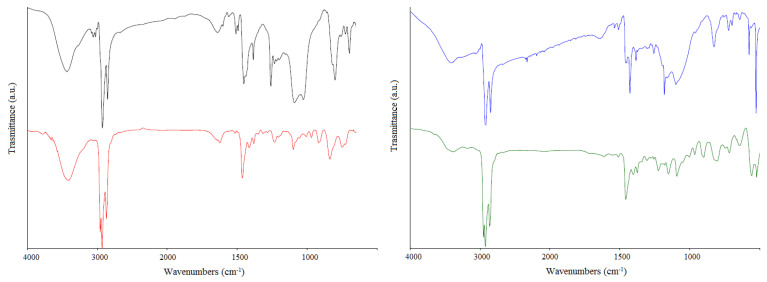
IR spectra of PT6Br (black line), PT6buP^+^ (red line), P[(T6Br)-*co*-(T6F)] (blue line) and P[(T6buP^+^)-*co*-(T6F)] (green line).

The IR spectra (Figure 3) of brominated derivatives show peaks, respectively, at 641, 558 cm^−1^ for PT6Br (black line) and 644, 557 cm^−1^ for P[(T6Br)-*co*-(T6F)] (blue line), which are characteristic of the terminal -Br atom in the side chain. The desired post-functionalization with C_60_-fullerene in the side chains is then confirmed by the presence of the absorptions at 1428, 1181, 562, and 526 cm^−1^ in P[(T6Br)-*co*-(T6F)] and at 1427, 1180, 576 and 526 cm^−1^ in P[(T6buP^+^)-*co*-(T6F)] (green line). On the other hand, the appearance of the bands at 2957, 2929, 2870, 1463, and 1378 cm^−1^ in PT6buP^+^ (red line) and at 2956, 2927, 2869, 1461, and 1379 cm^−1^ in P[(T6buP^+^)-*co*-(T6F)] spectra, which can be ascribed to the tributylphosphonium group, as well as the disappearance of the bands related to C-Br stretching evidence the complete post-functionalization of the polymeric precursors.

### 3.4. UV–Vis Analysis

**Figure 4:**
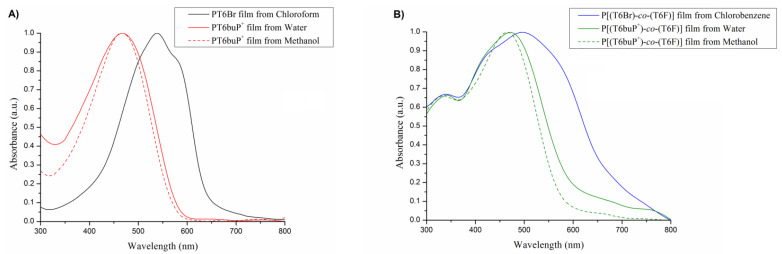
UV–Vis spectra of homo- (**A**) and double-cable copolymers (**B**) in thin film by drop-casting from different solvents.

The optical properties of PT6buP^+^ and P[(T6buP^+^)-*co*-(T6F)] were analyzed by UV–Vis spectroscopy in the solid state, by drop-casting their solution onto quartz slides, and subsequently compared with the respective polymeric precursor PT6Br and P[(T6Br)-*co*-(T6F)] films. Their spectra and assignments are shown in Figure 4 and Table 2.

Both spectra of PT6buP^+^ films (Figure 4A), obtained from methanol and water solutions, show similar broad bands with the maximum absorption wavelength (λ_max_) at 467 nm, ascribable to the π-π* electronic transition that typically occurs in the conjugated polymer main chain. However, the λ_max_ of PT6buP^+^ is strongly blue-shifted compared to that of the precursor PT6Br (Δλ _max_ = 71 nm); this fact could be related to the higher steric hindrance of the phosphonium group in the side chain, which prevents an efficient π-π stacking of the polymeric backbones and reduces the extension of the conjugated system.

On the other hand, the spectra of both double-cable copolymers in the solid state (Figure 4B) show behaviors quite different from those of the previously discussed homopolymers. In fact, due to the bulky and hindering nature of the fullerene units inserted in the side chain, whose absorption contribution is clearly evident at about ~340 nm, a rather strong blue-shift of the λ_max_ of the thiophene system is observed for P[(T6Br)-*co*-(T6F)]. Moreover, when the post-functionalization with phosphine has occurred, the obtained copolymer P[(T6buP^+^)-*co*-(T6F)] shows absorption maxima in the 465–471 nm range. These values are comparable with those obtained for the ionic homopolymeric derivative which is completely devoid of fullerene groups. This behavior may be ascribed both to the high film formation ability and to the reorganization of the ionic copolymer in a more planar and ordered system when deposited from polar solvents.

### 3.5. Thermal Properties

The thermal stability of homo- and double-cable copolymers was determined by TGA under oxidizing atmosphere (air), at a heating scan of 10 °C min^−1^ and up to 600 °C (Figure S13).

While in the TGA curve of PT6Br the two main weight losses can be ascribed to the elimination of HBr and side chains, for PT6buP^+^ the weight changes can be related to the loss of the phosphonium pendant and aliphatic side chain. In addition, the TGA curve of the ionic homopolymer also indicates a negligible loss of weight at around 80–100 °C, which may be ascribed to a residual water release, due to the strong hydrophilicity of PT6buP^+^. Overall, the homo-polymeric main chains are stable up to around 500 °C, since the last weight loss can be found at around 550 °C for both of them.

On the other hand, the double-cable copolymer’s thermal behavior is quite different from that of the respective polymeric precursors, and this may be ascribed to the steric hindrance of fullerene linked to the side chain. Indeed, even though a small weight loss at around 155 °C is shown in the P[(T6buP^+^)-*co*-(T6F)] sample, which is probably ascribable to solvent traces, it is possible to see two main weight losses instead of three, with different degradation temperatures, compared to the two homo-polymers (Table 3). The synthesized samples, however, may be considered stable enough to be used as photoactive materials in organic photovoltaic devices.

The DSC analysis was carried out under nitrogen atmosphere with a heating ramp of 10 °C min^−1^ from −20 °C up to 200 °C (Figure S14). All polymeric samples show an endothermic flexure, ascribable to the glass transition temperature (T_g_), a value that decreases when the tributylphosphonium group is linked to the side chain. Moreover, the presence of this ionic side chain moiety determines the absence of melting peaks in DSC curves of PT6buP^+^ and P[(T6buP^+^)-*co*-(T6F)].

### 3.6. X-ray Diffraction

**Figure 5:**
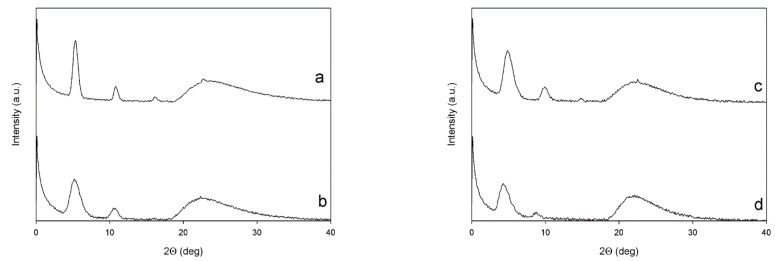
X-ray diffractograms of PT6Br (**a**), PT6buP^+^ (**b**), P[(T6Br)-*co*-(T6F)] (**c**) and P[(T6buP^+^)-*co*-(T6F)] (**d**).

The X-ray diffraction (XRD) profiles of homopolymers and copolymers in film are shown in Figure 5.

Samples exhibit the (100) reflection ascribable to the side chain lamellar distance and the corresponding high order reflection (200). The third order reflection (300) is only present in PT6Br and P[(T6Br)-*co*-(T6F)] diffractograms (Table 4).

The weak peaks at wide angles superimposed to the broad amorphous halo correspond to the on-plain chain distances. The lamellar spacings and the planes stacking distances range from 3.92 to 4.09 Å and 16.5 to 20.3 Å, respectively. The crystallites’ mean size (L), calculated using Scherrer’s relation [37], indicates the presence of some microcrystalline domains in the essentially amorphous phase of the examined polymeric films. The presence of the less cumbersome functional group (–Br) in the side chain has a positive effect on PT6Br behavior, determining the sharpening of the low-angle diffraction peaks, a better appearance of the high-angle peak and the formation of wider crystallites when compared to the other samples.

### 3.7. Photovoltaic Properties

Water-soluble polymers PT6buP^+^ and P[(T6buP^+^)-*co*-(T6F)] were tested as photoactive layers for the building up of BHJ solar cells. Photovoltaic properties were investigated by adopting the device configuration ITO/PEDOT:PSS/photoactive layer/Al. PT6buP^+^ was used in a 1:1 weight ratio (1:0.4 molar ratio) blend with C_60_-Ser, while P[(T6buP^+^)-*co*-(T6F)] was tested as a single material. Photovoltaic performance parameters are shown in Table 5.

**Figure 6:**
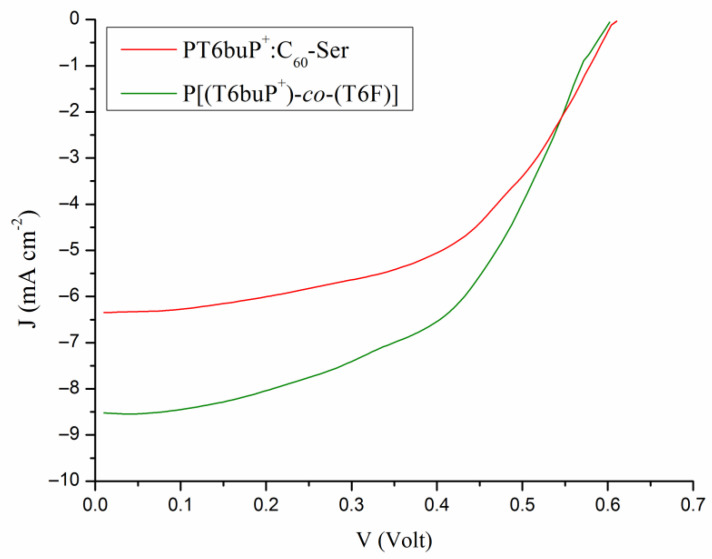
Current density–voltage for the best-performing cells tested under AM 1.5 one-sun illumination.

The photovoltaic parameters and current density–voltage curves shown in Figure 6 show that the best performance can be obtained using the double-cable copolymer. This material is characterized by a higher photo-current than its homopolymeric counterpart, and this may be ascribed to the presence of the fullerene group chemically linked to the polyconjugated backbone, thus avoiding phase-separation and aggregation phenomena usually observed when the electron acceptor molecule is only physically blended with the electron donor polymer. This result is particularly promising since, with the proposed approach, electron donor and electron acceptor moieties can be collected within a single material which, at the same time, can be applied to different surfaces, avoiding the use of toxic or non-environmentally friendly solvents.

### 3.8. EQE Measurements

**Figure 7:**
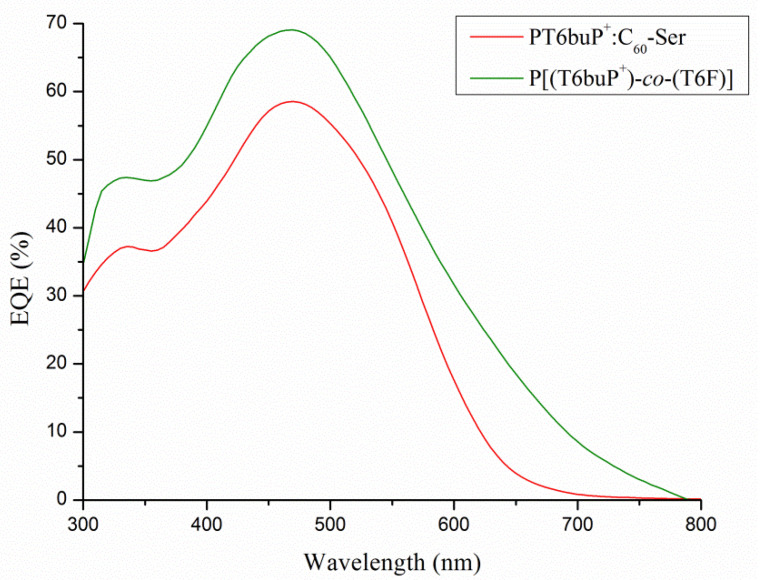
EQE spectra of homo- (PT6buP^+^) and co-polymer (P[(T6buP^+^)-*co*-(T6F)]) devices.

Photo-current response wavelengths for the cells based on the PT6buP^+^:C_60_-Ser photoactive blend and on the P[(T6buP^+^)-*co*-(T6F)] layer range from 300 to 790 nm and 300 to 700 nm, respectively. The relevant curves are shown in Figure 7, and the J_SC_ values, determined by EQE analysis, are shown in Table 5.

The curve of the device based on the PT6buP^+^:C_60_-Ser blend has two prominent peaks at 475 nm and 335 nm, while the device prepared with P[(T6buP^+^)-*co*-(T6F)] shows two feature peaks at 470 nm and 335 nm. Quantum efficiency (EQE) profiles follow the trend observed in the absorption spectra of homo- and copolymers in film. This suggests that the harvested photons over the whole absorption spectrum can contribute to the final photo-current. Moreover, an enhanced EQE is observed in the device made with P[(T6buP^+^)-*co*-(T6F)] copolymer (EQE_max_ 69%) compared to the photoactive physical blend (EQE_max_ 59%). According to the higher value of J_SC_ recorded for the device based on the double-cable copolymer, the obtained results indicate an improved charge collection efficiency when the electron acceptor material is directly linked to the electron donor main chain.

### 3.9. Morphological Analysis

**Figure 8:**
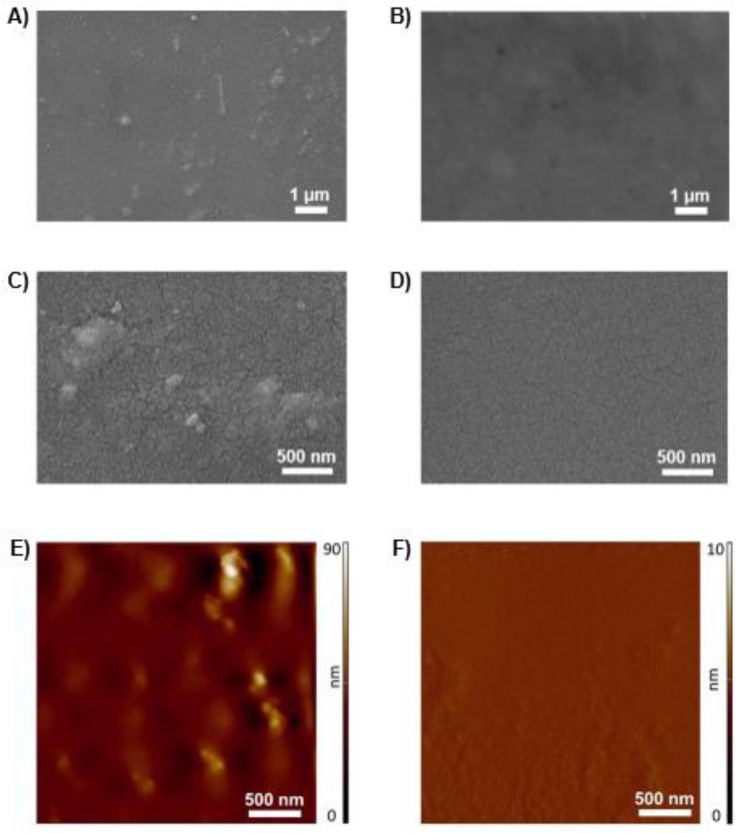
Morphological structure of the active layer surface of the organic photovoltaic cells. FE-SEM (**A**–**D**) and AFM topographical images (**E**,**F**) of the active material layers obtained from PT6buP^+^:C_60_-Ser (left column) and P[(T6buP^+^)-*co*-(T6F)] (right column).

The morphological features of the active layers made by PT6buP^+^:C_60_-Ser and P[(T6buP^+^)-*co*-(T6F)] were analyzed by FE-SEM (Figure 8A–D) and AFM (Figure 8E,F). Figure 8A,C show the high-resolution electron microscopy images of an active layer made by PT6buP^+^:C_60_-Ser. When the physical blend is used, the polymer layer microstructure and nanostructure show the typical aggregation pattern of fullerene-containing bulk heterojunction polymer films. The entire surface is affected by the formation of micrometric aggregates. Additionally, the presence of aggregates is evident even at the nanoscale in Figure 8C. This phenomenon, which is usually associated with the typical efficiency drop of BHJ solar cells, is confirmed by the results obtained by analyzing this kind of active layer by AFM (Figure 8E). As it is clear by observing FE-SEM images (Figure 8B,D) as well as the AFM topography (Figure 8F), the active layer of the single-material based cell obtained using the synthesized double-cable polymer is extremely flat and does not show any micro- or nanoscale defect or tendency to form aggregates. The results collected by using these high-resolution imaging techniques confirm the absence of phase separation and the outstanding uniformity of the P[(T6buP^+^)-*co*-(T6F)] structure and justify the best photovoltaic performances obtained with this sample. The uniformity, stability, and absence of imperfections reveal an enhanced hierarchical nano- and microstructuration of the single-material double-cable layer driven by the proposed elegant chemical structure optimization, which is undoubtedly vital for the development of highly efficient single-material organic photovoltaic solar cells.

## 4. Conclusions

Two tributylphosphine-functionalized polyalkylthiophenes were synthesized with the aim of preparing new water-soluble photoactive layers for organic solar cells. A new double-cable donor–acceptor polythiophene derivative has been successfully synthesized using an easy and effective post-polymerization approach on regioregular poly[3-(6-bromohexylthiophene)] (PT6Br) by means of a Grignard coupling reaction with C_60_-fullerene. The successful anchorage of C_60_ to the PT6Br backbone was confirmed by FT-IR and NMR analysis, which also made it possible to determine the substitution degree. The obtained copolymer was further functionalized with tributylphosphine in order to enhance its solubility in polar solvents. The final material showed good solubility in water, as well as methanol, and was successfully used as a single-material photoactive layer in organic photovoltaic solar cells. Its photoconversion performance was compared with that measured using a device exploiting a water-soluble photoactive blend prepared with a tributylphosphine-substituted regioregular poly(3-hexylthiophene) and a C_60_-serinol derivative. The copolymer sample showed the most performing properties, confirming the pivotal effect of the chemical structure on the electronic characteristics of the photoactive blend. Indeed, the EA group’s presence directly linked to the polythiophenic ED backbone can reduce the distance between the two electroactive systems, making possible a more effective exciton dissociation and a faster charge-transport to the electrodes. The suggested synthetic approach—leading to a single copolymeric electron donor material which contains both water-solubilizing groups and electron acceptor moieties at the same time—provides a new simple and versatile architecture for the construction of efficient donor–acceptor intramolecular polymers. Indeed, the reported method can be further exploited for the assembly of green water-soluble single-material solar cells.

## Figures and Tables

**Table 1:** Polymers’ characteristics.

	Reaction Yield (%)	Ionic Group Content (%mol) ^a^	C_60_Content (%mol) ^a^	Mn (kDa)	PDI
PT6Br	21	-	-	14.9 ^b^	1.15 ^b^
PT6buP^+^	98	100	-	27.2 ^c^	1.15 ^c^
P[(T6Br)-*co*-(T6F)]	85	-	7	17.6 ^c^	1.15 ^c^
P[(T6buP^+^)-*co*-(T6F)]	78	93	7	29.1 ^c^	1.15 ^c^

^a^ Determined by ^1^H-NMR; ^b^ Determined by GPC relative to polystyrene standards; ^c^ Calculated from the molecular mass of the corresponding homo- (PT6Br) and co-polymeric (P[(T6Br)-*co*-(T6F)]) precursors.

**Table 2:** Maximum absorption wavelengths of polymers in thin films.

	Solvents	λ_max_ Film (nm)
PT6Br	Chloroform	538
PT6buP^+^	Water	467
	Methanol	467
P[(T6Br)-*co*-(T6F)]	Chlorobenzene	340, 496
P[(T6buP^+^)-*co*-(T6F)]	Water	343, 471
	Methanol	334, 465

**Table 3:** Glass-transition (T_g_), melting (T_m_), crystallization (T_c_) and initial decomposition (T_d_) temperatures of polymers.

Sample	T_g_ (°C)	T_m_ (°C)	T_c_ (°C)	T_d_ (°C)
PT6Br	55	164	95	282
PT6buP^+^	43	-	42	355
P[(T6Br)-*co*-(T6F)]	77	148	86	260
P[(T6buP^+^)-*co*-(T6F)]	27	-	22	296

**Table 4:** Structural parameters of samples.

Sample	Low-Angle Diffractions	High-Angle Diffractions	On Plane Th Chains Distances	Planes Stacking Distances	Crystallites Mean Size (L)
	(Degrees)	(Degrees)	(Å)	(Å)	(nm)
PT6Br	5.34; 10.82; 16.19	22.68	16.5	3.92	15.6
PT6buP^+^	5.24; 10.61	22.34	16.8	3.98	6.61
P[(T6Br)-*co*-(T6F)]	4.91; 9.89; 14.84	22.51	17.9	3.95	6.74
P[(T6buP^+^)-*co*-(T6F)]	4.34; 8.71	21.61	20.3	4.09	5.33

**Table 5:** Short-circuit current density (J_SC_), open-circuit voltage (V_OC_), fill factor (FF) and power conversion efficiency (PCE) of tested BHJ solar cells. Average performances are taken from ten devices.

Sample	J_SC_ (mA cm^−2^) ^(a)^	V_OC_ (V)	FF	PCE (%)	J_SC_ (mA cm^−2^) ^(b)^
PT6buP^+^:C_60_-Ser	6.36 ± 0.3	0.61 ± 0.01	0.59 ± 0.04	2.29 ± 0.13	6.22
P[(T6buP^+^)-*co*-(T6F)]	8.51 ± 0.4	0.61 ± 0.01	0.60 ± 0.05	3.11 ± 0.15	8.78

^(a)^ From J/V curves; ^(b)^ From EQE measurements.

## Data Availability

The data that support the findings of this study are available from the corresponding author.

## Supplementary Materials

The following are available online at https://www.mdpi.com/article/10.3390/polym13101640/s1, Figure S1: ^1^H-NMR spectrum of PT6buP^+^, Figure S2: ^13^C-NMR spectrum of PT6buP^+^, Figure S3: ^13^C-NMR of P[(T6buP^+^)-*co*-(T6F)], Figure S4: ^13^C-NMR spectrum of PT6Br, Figure S5: ^13^C-NMR spectrum of P[(T6Br)-*co*-(T6F)], Figure S6: ^31^P-NMR spectra of PT6buP^+^ and P[(T6buP^+^)-*co*-(T6F)], Figure S7: ^1^H-NMR spectrum of P-Ser, Figure S8: ^1^H-NMR spectrum of PC_60_-Ser, Figure S9: ^1^H-NMR spectrum of C_60_-Ser, Figure S10: ^13^C-NMR of P-Ser, Figure S11: ^13^C-NMR of PC_60_-Ser, Figure S12: ^13^C-NMR of C_60_-Ser, Figure S13: TGA thermograms of homo- (PT6Br and PT6buP^+^) and *co*-polymers (P[(T6Br)-co-(T6F)] and P[(T6buP+)-co-(T6F)]), Figure S14: DSC thermograms of homo- (PT6Br and PT6buP^+^) and co-polymers (P[(T6Br)-*co*-(T6F)] and P[(T6buP+)-*co*-(T6F)]). Table S1: Study of PT6Br synthesis by GRIM reaction: chemical shifts and assignments of the three samples collected at different times, Table S2: Main IR absorption bands of homo-polymers and double-cable copolymers, Table S3: Main IR absorption bands of P-Ser, PC_60_-Ser and C_60_-Ser.

## References

[1] Mazzio K.A., Luscombe C.K. (2015). The future of organic photovoltaics. Chem. Soc. Rev..

[2] Yip H.-L., Jen A.K.-Y. (2012). Recent advances in solution-processed interfacial materials for efficient and stable polymer solar cells. Energy Environ. Sci..

[3] Lu L., Zheng T., Wu Q., Schneider A.M., Zhao D., Yu L. (2015). Recent Advances in Bulk Heterojunction Polymer Solar Cells. Chem. Rev..

[4] Lanzi M., Salatelli E., Benelli T., Caretti D., Giorgini L., Di-Nicola F.P. (2015). A regioregular polythiophene-fullerene for polymeric solar cells. J. Appl. Polym. Sci..

[5] Lanzi M., Salatelli E., Marinelli M., Pierini F. (2019). Effect of Photocrosslinking of D-A Thiophene Copolymers on the Performance of Single-Material Solar Cells. Macromol. Chem. Phys..

[6] Das S., Chatterjee D.P., Ghosh R., Nandi A.K. (2015). Water soluble polythiophenes: Preparation and applications. RSC Adv..

[7] Duan C., Zhang K., Zhong C., Huang F., Cao Y. (2013). Recent advances in water/alcohol-soluble π-conjugated materials: New materials and growing applications in solar cells. Chem. Soc. Rev..

[8] Lanzi M., Salatelli E., Giorgini L., Mucci A., Pierini F., Di-Nicola F.P. (2017). Water-soluble polythiophenes as efficient charge-transport layers for the improvement of photovoltaic performance in bulk heterojunction polymeric solar cells. Eur. Polym. J..

[9] Huang F., Wu H., Cao Y. (2010). Water/alcohol soluble conjugated polymers as highly efficient electron transporting/injection layer in optoelectronic devices. Chem. Soc. Rev..

[10] Lanzi M., Salatelli E., Giorgini L., Marinelli M., Pierini F. (2018). Effect of the incorporation of an Ag nanoparticle interlayer on the photovoltaic performance of green bulk heterojunction water-soluble polythiophene solar cells. Polymer.

[11] Ghoos T., Brassinne J., Fustin C.-A., Gohy J.-F., Defour M., Brande N.V.D., Van Mele B., Lutsen L., Vanderzande D.J., Maes W. (2013). Imidazolium-substituted ionic (co)polythiophenes: Compositional influence on solution behavior and thermal properties. Polymer.

[12] Urbanek P., Di Martino A., Gladyš S., Kuritka I., Minařik A., Pavlova E., Bondarev D., Hladysh S. (2015). Polythiophene-based conjugated polyelectrolyte: Optical properties and association behavior in solution. Synth. Met..

[13] Hladysh S., Murmiliuk A., Vohlídal J., Havlíček D., Sedlarik V., Stepanek M., Zednik J. (2018). Combination of phosphonium and ammonium pendant groups in cationic conjugated polyelectrolytes based on regioregular poly(3-hexylthiophene) polymer chains. Eur. Polym. J..

[14] Hladysh S., Bondarev D., Svoboda J., Vohlídal J., Vrbata D., Zedník J. (2016). Novel conjugated polyelectrolytes based on polythiophene bearing phosphonium side groups. Eur. Polym. J..

[15] López A.M., Mateo-Alonso A., Prato M. (2010). Materials chemistry of fullerene C60derivatives. J. Mater. Chem..

[16] Markovic Z., Trajkovic V. (2008). Biomedical potential of the reactive oxygen species generation and quenching by fullerenes (C60). Biomaterials.

[17] Kokubo K., Matsubayashi K., Tategaki H., Takada H., Oshima T. (2008). Facile Synthesis of Highly Water-Soluble Fullerenes More Than Half-Covered by Hydroxyl Groups. ACS Nano.

[18] Li H., Haque S.A., Kitaygorodskiy A., Meziani M.J., Torres-Castillo M., Sun Y.-P. (2006). Alternatively Modified Bingel Reaction for Efficient Syntheses of C60Hexakis- Adducts. Org. Lett..

[19] Lanzi M., Costa-Bizzarri P., Paganin L., Cesari G. (2007). Synthesis by post-polymerization functionalization of sensitive polythiophenes for selective chemo-recognition purposes. React. Funct. Polym..

[20] Mitchell R.H., Lai Y.-H., Williams R.V. (1979). N-Bromosuccinimide-dimethylformamide: A mild, selective nuclear monobromination reagent for reactive aromatic compounds. J. Org. Chem..

[21] McCullough R.D. (1998). The Chemistry of Conducting Polythiophenes. Adv. Mater..

[22] Stefan M.C., Bhatt M.P., Sista P., Magurudeniya H.D. (2011). Grignard metathesis (GRIM) polymerization for the synthesis of conjugated block copolymers containing regioregular poly(3-hexylthiophene). Polym. Chem..

[23] Loewe R.S., Ewbank P.C., Liu J., Zhai A.L., McCullough R.D. (2001). Regioregular, Head-to-Tail Coupled Poly(3-alkylthiophenes) Made Easy by the GRIM Method: Investigation of the Reaction and the Origin of Regioselectivity. Macromolecules.

[24] Hoppe H., Sariciftci N.S. (2006). Morphology of polymer/fullerene bulk heterojunction solar cells. J. Mater. Chem..

[25] Hirsch A., Vostrowsky O. (2001). C_60_ Hexakisadducts with an Octahedral Addition Pattern—A New Structure Motif in Organic Chemistry. Eur. J. Org. Chem..

[26] Wharton T., Kini V.U., Mortis R.A., Wilson L.J. (2001). New non-ionic, highly water-soluble derivatives of C60 designed for biological compatibility. Tetrahedron Lett..

[27] Bar-Shir A., Engel Y., Gozin M. (2005). Synthesis and Water Solubility of Adamantyl-OEG-fullerene Hybrids. J. Org. Chem..

[28] Shirosaki T., Harisaki R., Horikawa M., Sakurai H., Nagaoka S., Ihara H. (2013). Synthesis of a Series of Malonic Diester–Introduced Fullerene Derivatives. Synth. Commun..

[29] Wharton T., Wilson L.J. (2002). Highly-Iodinated Fullerene as a Contrast Agent For X-ray Imaging. Bioorganic Med. Chem..

[30] Sheina E.E., Liu J., Iovu M.C., Laird D.W., McCullough R.D. (2004). Chain Growth Mechanism for Regioregular Nickel-Initiated Cross-Coupling Polymerizations. Macromolecules.

[31] Lohwasser R.H., Thelakkat M. (2011). Toward Perfect Control of End Groups and Polydispersity in Poly(3-hexylthiophene) via Catalyst Transfer Polymerization. Macromolecules.

[32] Chen T.-A., Wu X., Rieke R.D. (1995). Regiocontrolled Synthesis of Poly(3-alkylthiophenes) Mediated by Rieke Zinc: Their Characterization and Solid-State Properties. J. Am. Chem. Soc..

[33] Koch F.P.V., Smith P., Heeney M. (2013). “Fibonacci’s Route” to Regioregular Oligo(3-hexylthiophene)s. J. Am. Chem. Soc..

[34] Iovu M.C., Sheina E.E., Gil R.R., McCullough R.D. (2005). Experimental Evidence for the Quasi-“Living” Nature of the Grignard Metathesis Method for the Synthesis of Regioregular Poly(3-alkylthiophenes). Macromolecules.

[35] Zagórska M., Krische B. (1990). Chemical synthesis and characterization of soluble poly(4,4′-dialkyl-2,2′-bithiophenes). Polymer.

[36] Jeffries-El M., Sauvé G., McCullough R.D. (2005). Facile Synthesis of End-Functionalized Regioregular Poly(3-alkylthiophene)s via Modified Grignard Metathesis Reaction. Macromolecules.

[37] Mayer A.C., Toney M.F., Scully S.R., Rivnay J., Brabec C.J., Scharber M., Koppe M., Heeney M., McCulloch I., McGehee M.D. (2009). Bimolecular Crystals of Fullerenes in Conjugated Polymers and the Implications of Molecular Mixing for Solar Cells. Adv. Funct. Mater..

