# Supplementary material for: Influence of the Active Layer Structure on the Photovoltaic Performance of Water-Soluble Polythiophene-Based Solar Cells
Source: Polymers (Basel). 2021 May 18;13(10):1640. doi: 10.3390/polym13101640 (PMC8158483; doi:10.3390/polym13101640)
Supplement: Supplementary file 1 [file polymers-13-01640-s001.zip › polymers-1214530-supplementary.pdf]

polymers

MDPI

Supplementary Materials

Influence of the Active Layer Structure on the Photovoltaic Performance of Water-Soluble Polythiophene-Based Solar Cells

Massimiliano Lanzi [1,2*], Debora Quadretti [1], Martina Marinelli [1,3], Yasamin Ziai [3], Elisabetta Salatelli [1] and Filippo Pierini [3]

[1] Department of Industrial Chemistry "Toso Montanari", University of Bologna, Viale Risorgimento 4, 40136 Bologna, Italy; debora.quadretti2@unibo.it (D.Q.); martina.marinelli5@unibo.it (M.M.); elisabetta.salatelli@unibo.it (E.S.)
[2] INSTM-National Interuniversity Consortium of Materials Science and Technology, Via G. Giusti 9, 50121 Firenze, Italy; massimiliano.lanzi@unibo.it (M.L.)
[3] Department of Biosystem and Soft Matter, Institute of Fundamental Technological Research, IPPT-PAN, Polish Academy of Science, ul. Pawinskiego 5B, 02-106 Warsaw, Poland; yziai@ippt.pan.pl (Y.Z.); fpierini@ippt.pan.pl (F.P.)
* Correspondence: massimiliano.lanzi@unibo.it

Table S1. Study of PT6Br synthesis by GRIM reaction: chemical shift (ppm) and assignments of the three samples collected at different times.

A (*30 min*)	*B* (*60 min*)	*C* (*90 min*)	*Assignment*	*Ref.*
7.19	7.19	-	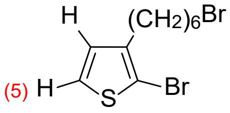	[31]
-	7.00	-	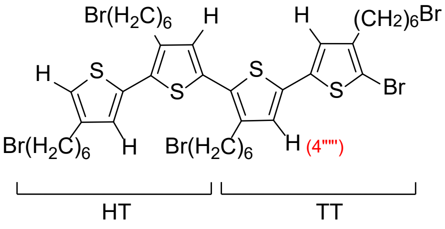	
-	6.98	6.98	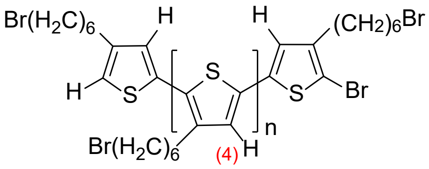	[32]
-	6.91	-	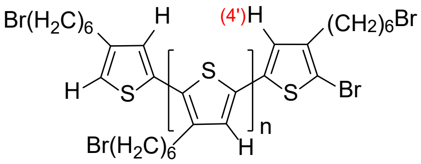	[33]
6.88	6.88	-	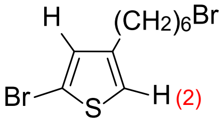	[31]

-	6.87	-	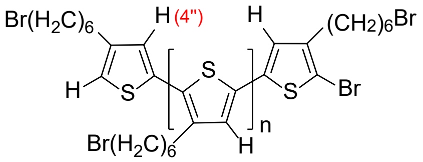	[34]
-	6.81	-	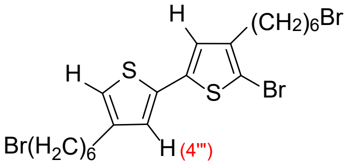	[35,36]
6.79	6.79	-	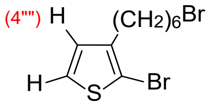	[31]
-	-	3.43	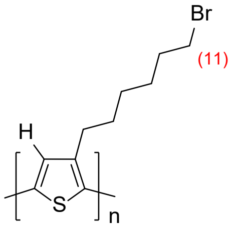	
3.41	3.41		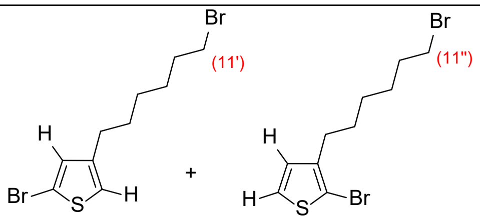	
-	2.83	2.83	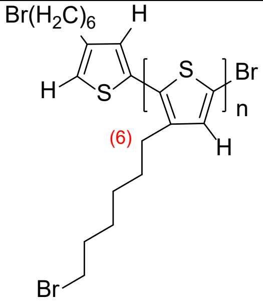	[32]
-	2.65	-	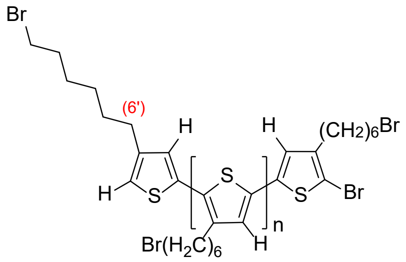	[33,34]

-	2.39	-	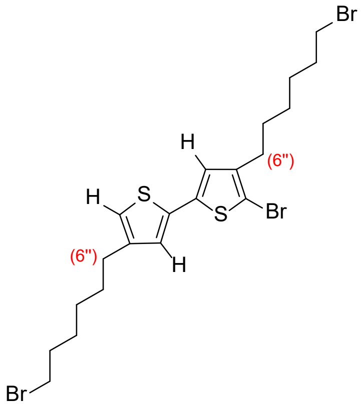	
1.86	1.86	1.90	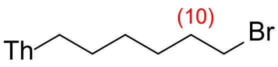	
1.56	1.56, 1.73	1.73	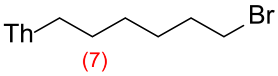	
1.47	1.47	1.49	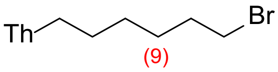	
1.36	1.36	1.49	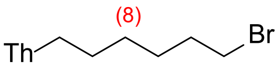	

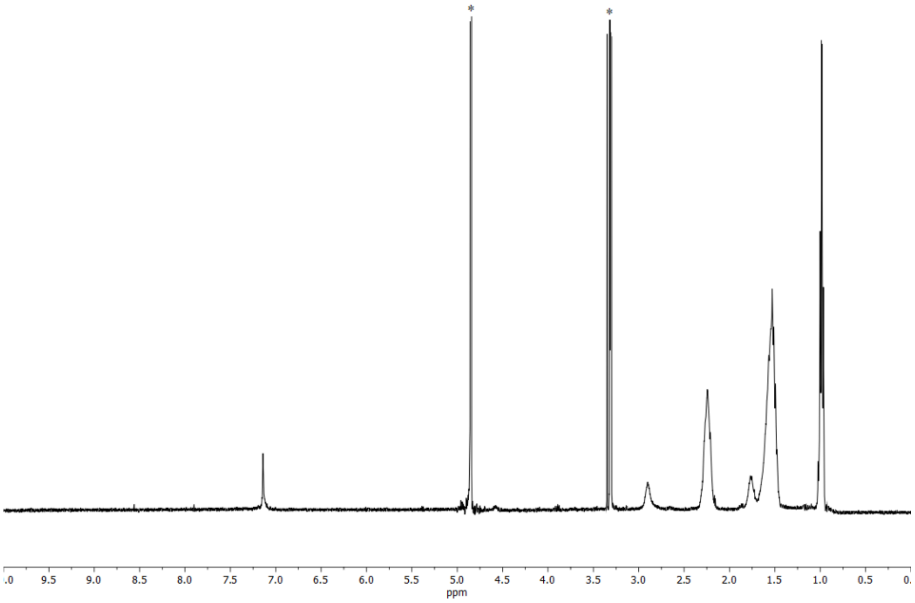

Figure S1. ^{1}H-NMR spectrum of PT6buP$^+$. Asterisk: solvent resonance (CD_3OD).

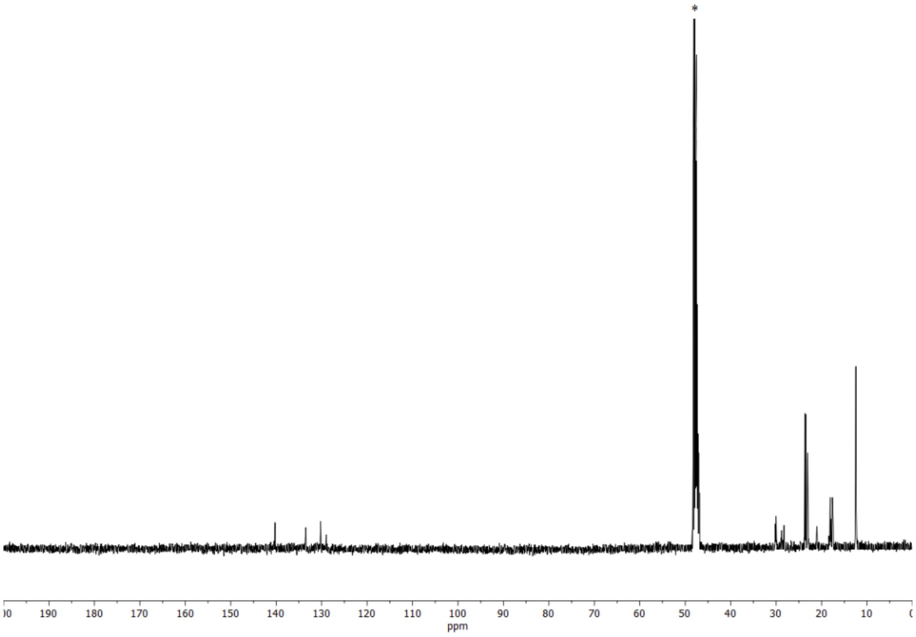

Figure S2. ^{13}C-NMR spectrum of $PT6buP^+$. Asterisk: solvent resonance (CD_3OD).

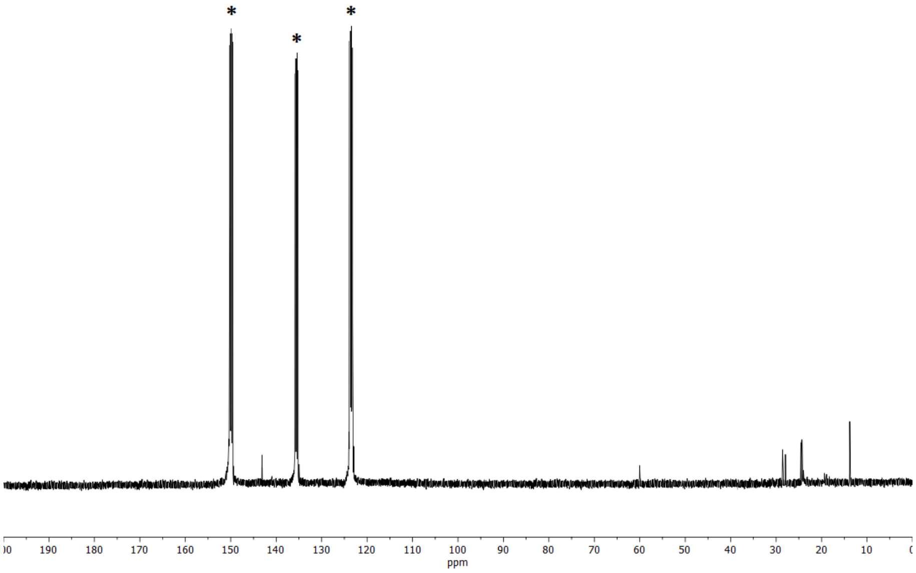

Figure S3. ^{13}C-NMR of P[(T6buP$^+$)-*co*-(T6F)]. Asterisk: solvent resonance (pyridine-d_5).

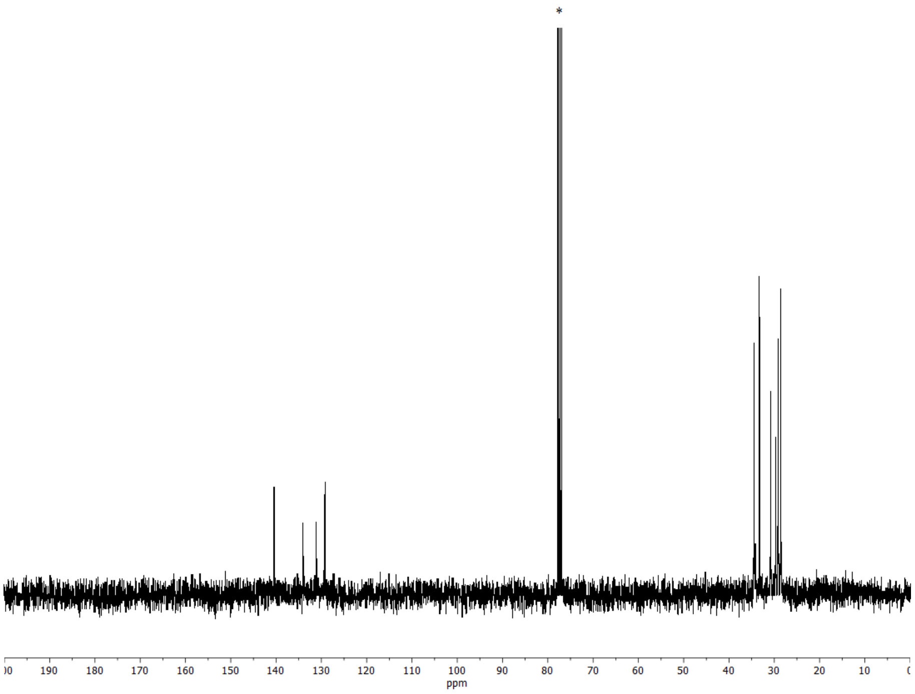

Figure S4. ^{13}C-NMR spectrum of PT6Br. Asterisk: solvent resonance ($CDCl_3$).

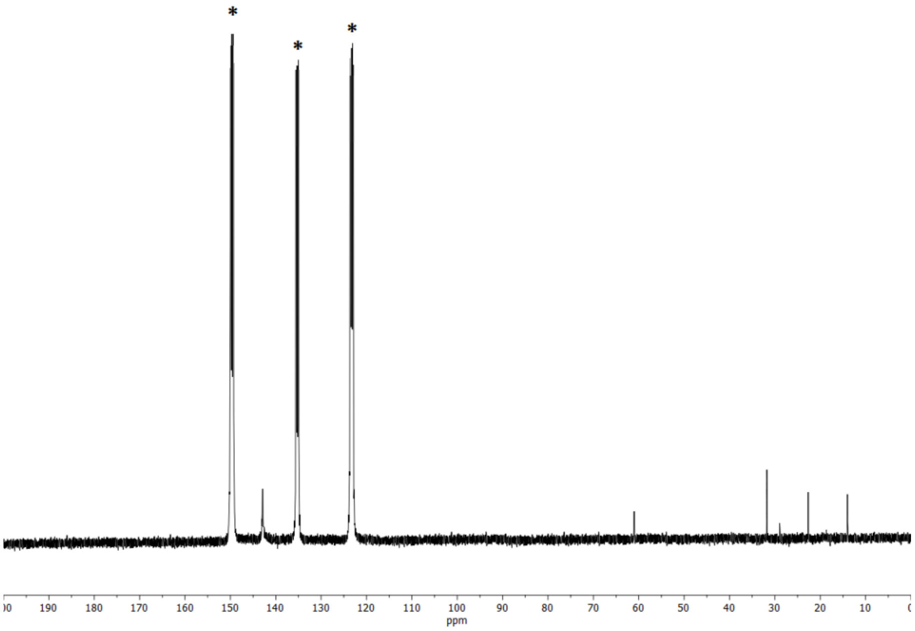

Figure S5. ^{13}C-NMR spectrum of P[(T6Br)-*co*-(T6F)]. Asterisk: solvent resonance (pyridine-d_5).

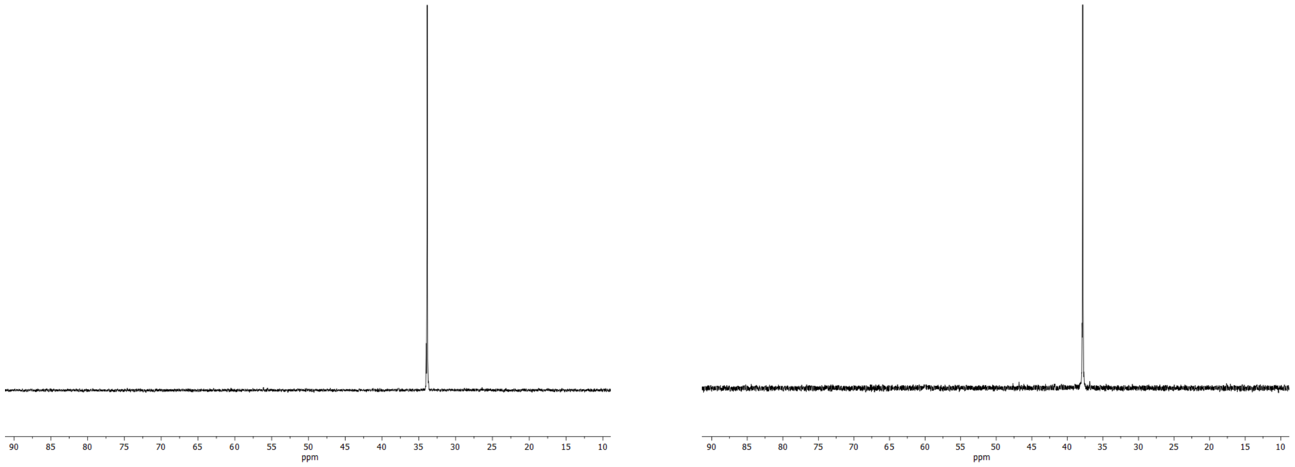

Figure S6. ^{31}P-NMR spectra of PT6buP$^+$ (left) and P[(T6buP$^+$)-*co*-(T6F)] (right) recorded in CD_3OD.

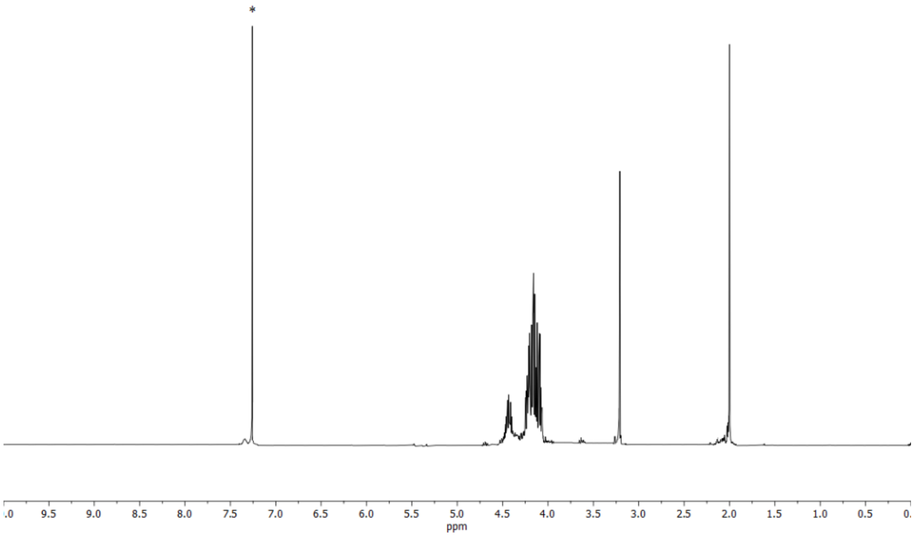

Figure S7. ^{1}H-NMR spectrum of P-Ser. Asterisk: solvent resonance ($CDCl_3$).

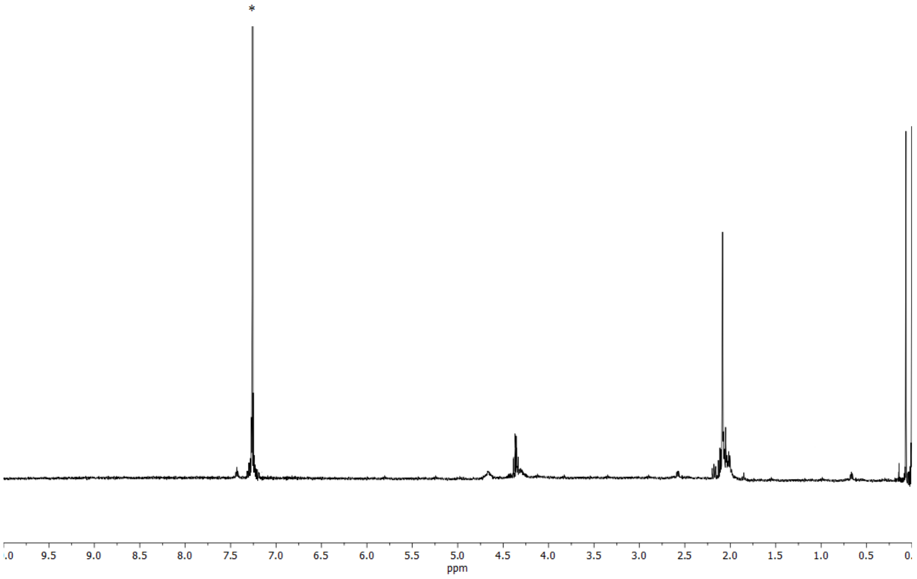

Figure S8. ^{1}H-NMR spectrum of PC_{60}-Ser. Asterisk: solvent resonance ($CDCl_3$).

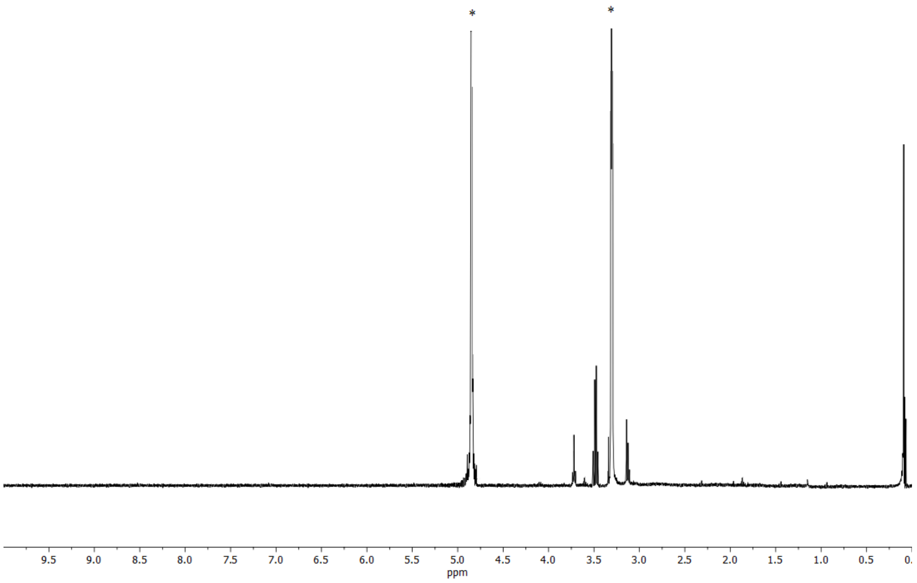

Figure S9. ^{1}H-NMR spectrum of C_{60}-Ser. Asterisk: solvent resonance (CD_3OD).

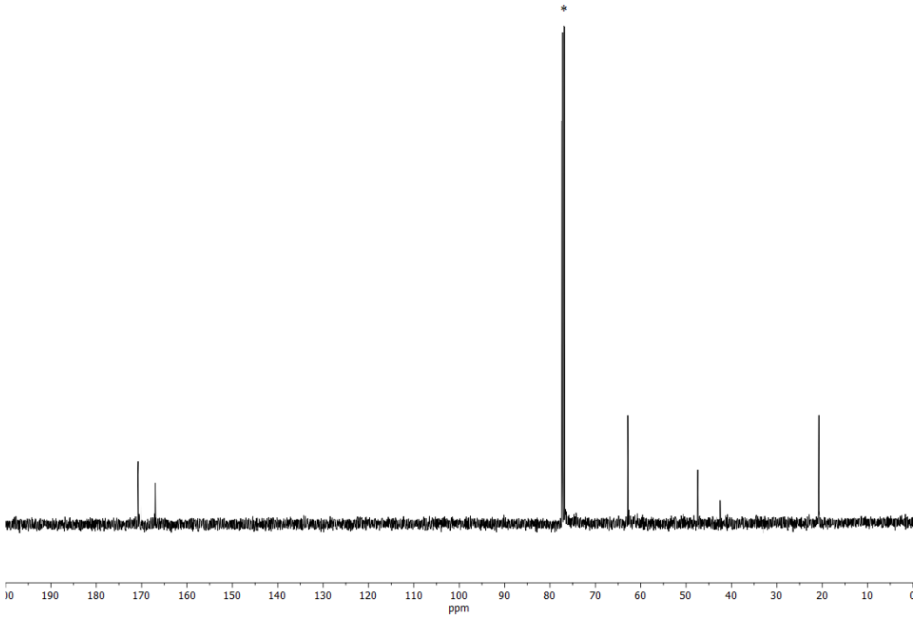

Figure S10. ^{13}C-NMR of P-Ser. Asterisk: solvent resonance ($CDCl_3$).

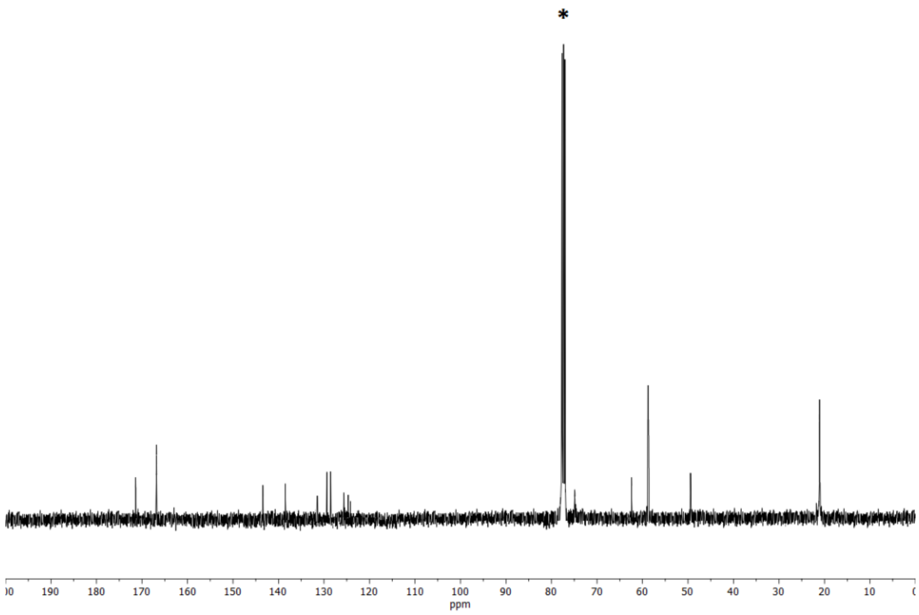

Figure S11. ^{13}C-NMR of PC_{60}-Ser. Asterisk: solvent resonance ().

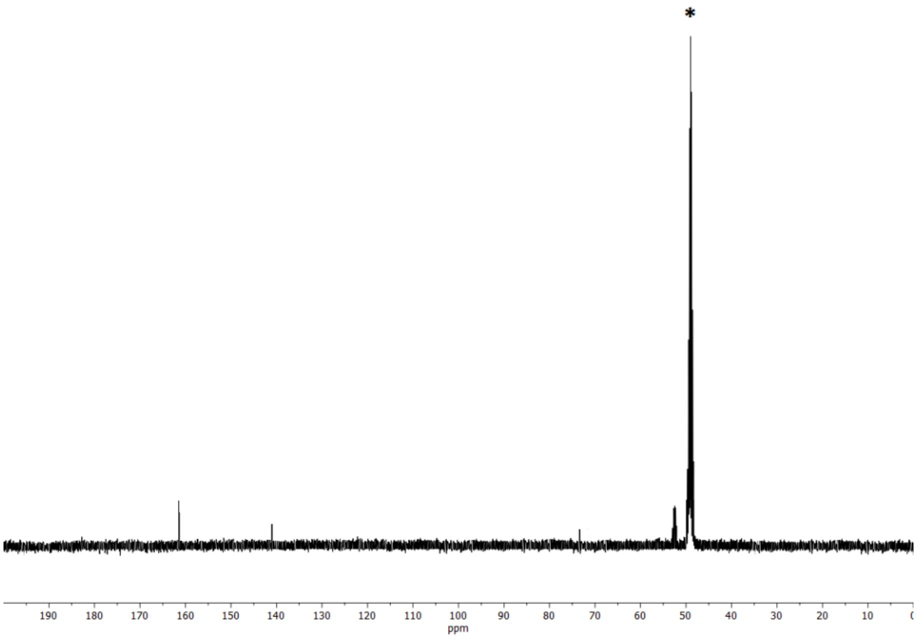

Figure S12. ^{13}C-NMR of C_{60}-Ser. Asterisk: solvent resonance ().

Table S2. Main IR absorption bands (cm^{-1}) of homo-polymers and double-cable copolymers.

PT6Br	***PT6buP$^+$***	***P[(T6Br)-co-(T6F)]***	***P[(T6buP$^+$)-co-(T6F)]***	***Assignment***
3058	3053	3053	3052	υ C-H β thiophene
-	2957	-	2956	υ_{as} –CH_3 (phosphonium group)
2923	2929	2918	2927	υ_{as} -CH_2- (side chain and phosphonium group)
2852	2870	2849	2869	υ_{sym} -CH_2- and –CH_3
1508	1513	1509	1514	υ_{as} -C=C- thiophene
1434	1463	1452	1461	-CH_2- deformation (phosphonium group) and υ_{sym} C=C thiophene
-	-	1428	1427	fullerene
-	1410	-	1408	γ_{as} P-CH_2-R
1384		1384		-CH_3 deformation
-	1378	-	1379	-CH_3 deformation (phosphonium group)
1259	1232	1257	1228	υ C-C thiophene-thiophene
-	-	1181	1180	fullerene
1089	1099	1099	1096	δ –CH thiophene
800	833	826	808	γ C-H thiophene 2, 3, 5-trisubstituted
725	723	722	721	rocking -CH_2-
641, 558	-	644, 557	-	*υ* C-Br aliphatic
-	-	562, 526	576, 526	fullerene

Table S3. Main IR absorption bands (cm^{-1}) of P-Ser, PC_{60}-Ser and C_{60}-Ser.

P-Ser	*PC₆₀-Ser*	*C₆₀-Ser*	*Assignment*
3301	3289	3405	υ N–H
2961	2926	2948	υ_{as} –CH_2–
2902	2853	2623	υ_{sym} –CH_2–
1737	1739	-	υ C=O ester
1661	1646	1649	Amide I band, υ C-O
1537	1543		Amide II band, δ N-H
-	1428	1429	fullerene
-	-	1401	γ OH
1370	1366	1371	Amide III band, υ C-N
1242	1228	-	υ_{as} –$OCOCH_3$
-	1180	1182	fullerene
-	561, 526	576, 526	fullerene

υ = stretching; γ = out of plane bending; δ = in-plane bending.

Thermal properties

TGA analyses

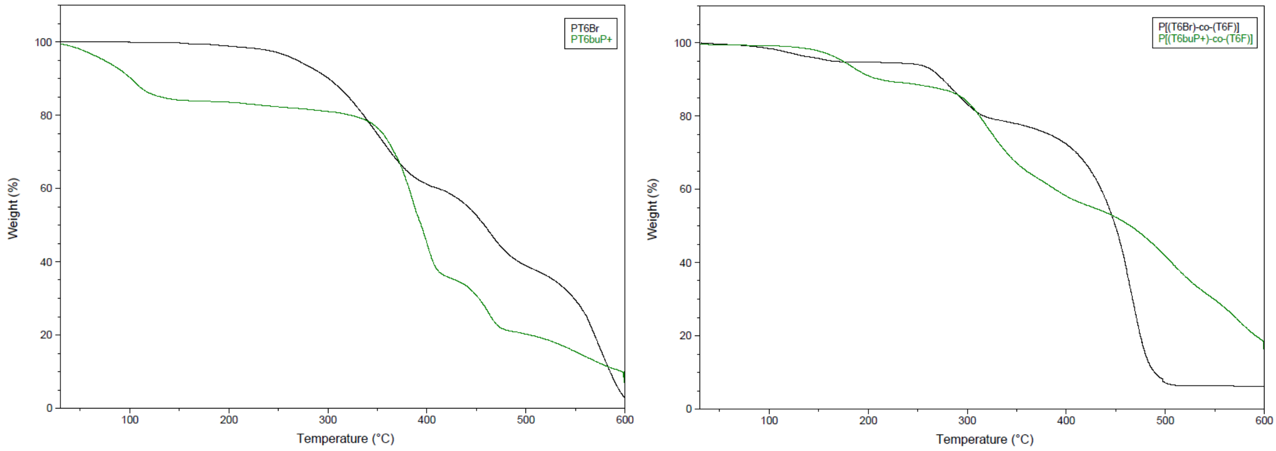

Figure S13. TGA thermograms of homo- (PT6Br and PT6buP⁺, left) and co-polymers (P[(T6Br)-*co*-(T6F)] and P[(T6buP⁺)-*co*-(T6F)], right).

DSC analyses

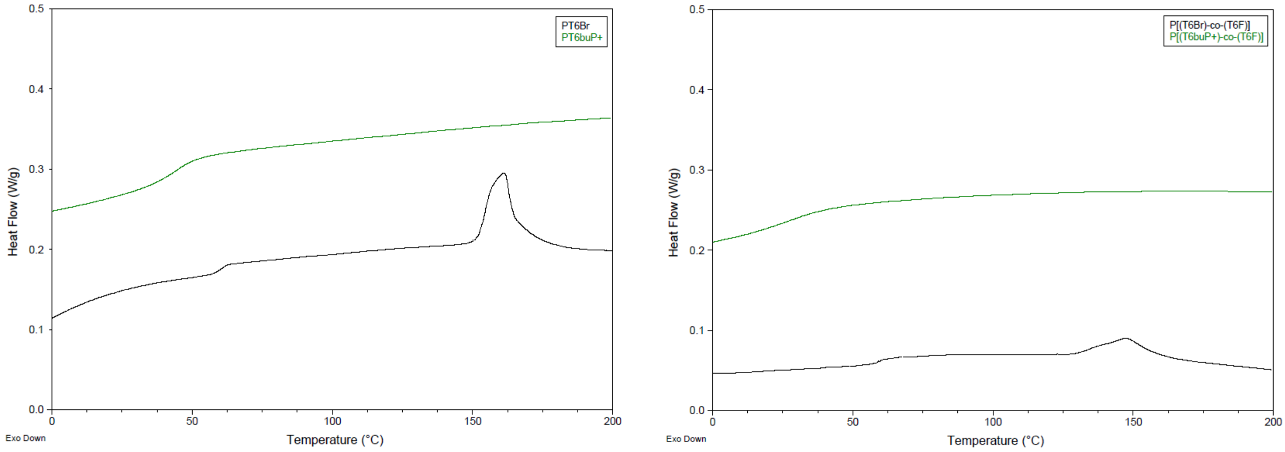

Figure S14. DSC thermograms of homo- (PT6Br and PT6buP⁺, left) and co-polymers (P[(T6Br)-*co*-(T6F)] and P[(T6buP⁺)-*co*-(T6F)], right).
